# Porphyrin-lipid nanotheranostics for multimodal imaging of nodal disease in preclinical oral cancers

**DOI:** 10.7150/thno.129853

**Published:** 2026-05-29

**Authors:** Michael S. Valic, Esmat Najjar, Mark Zheng, Jason L. Townson, Harley H. L. Chan, Sharon Tzelnick, Alessandra Ruaro, Abdullah El-Sayes, Michael Halim, Pamela Schimmer, Chris J. Zhang, Tina Ye, Wenlei Jiang, Juan Chen, Jonathan C. Irish, Gang Zheng

**Affiliations:** 1 Princess Margaret Cancer Centre, University Health Network, Toronto, Canada.; 2 Institute of Biomedical Engineering, Faculty of Applied Science and Engineering, University of Toronto, Toronto, Canada.; 3 Guided Therapeutics Program, University Health Network, Toronto, Canada.; 4 Department of Otolaryngology–Head and Neck Surgery, Temerty Faculty of Medicine, University of Toronto, Toronto, Canada.; 5 Department of Medical Biophysics, Temerty Faculty of Medicine, University of Toronto, Toronto, Canada.

**Keywords:** nanoparticles, radiotracers, fluorescence, lymph node metastasis, oral cancers

## Abstract

**Rationale:**

Cervical lymph node metastases in oral cancer patients are a frequent occurrence and important prognostic factor. Anatomical and molecular imaging modalities can identify neck metastases with varying sensitivity and specificity but perform poorly in clinically negative neck nodes with microscopic disease. Herein we investigate the use of porphyrin-lipid nanotheranostics (PS) for multimodal detection of neck disease in preclinical models of oral cancer.

**Methods:**

Xenograft models of tongue cancer were established in nude rats using MOC2 mouse oral squamous cells. PS nanoparticles were radiolabelled with positron-emitting Copper-64 (^64^Cu-PS) and administered either IT (100 MBq ^64^Cu, 0.5 mg) or IV (250–500 MBq ^64^Cu/kg, 0.5–1.0 mg/kg). Uptake in the tumour and cervical lymph nodes was measured with serial PET/MR imaging and at endpoint with *in situ* fluorescence (FL) imaging. In the IV cohort, ^64^Cu-PS uptake was compared to ^18^F-FDG (46 MBq ^18^F/kg) PET performed prior to nanoparticle injection. After imaging, neck nodes were harvested for pathological staging. Receiver operating characteristics were compared in the IV cohort between ^18^F-FDG *vs* ^64^Cu-PS PET imaging *vs* FL imaging.

**Results:**

The tongue tumour model yielded micrometastases to 54% of neck nodes by 14 d post-implantation. Following IT injection, sentinel and metastatic lymph nodes were visible with ^64^Cu-PS PET for 72 h. Following IV injection, ^64^Cu-PS PET signal in the tumour and neck nodes was clearest at 24 h and significant differences in the pooled SUVs of benign *vs* metastatic nodes were obtained. On FL imaging, metastatic neck nodes had significantly higher fluorescent signal (S/B) compared to benign nodes. Overall, FL S/B_mean_ gave the best prediction of nodal disease with 75% SEN, 79% SPC, and 71% NPV. ^64^Cu-PS PET (SUV_max_: 77% SEN, 68% SPC, 68% NPV) performed slightly worse than FL imaging for identifying metastatic nodes but still better compared to ^18^F-FDG PET (SUV_max_: 81% SEN, 43% SPC, 59% NPV). Inflamed nodes were the commonest source of false positives for both ^64^Cu-PS PET and FL imaging modalities.

**Conclusions:**

Nanotheranostic^ 64^Cu-PS permitted more accurate multimodal detection of microscopic neck disease in preclinical oral cancer models and can offer valuable guidance for planning and performing neck dissections.

## Introduction

Oral cancers are a family of diseases originating primarily from the thin squamous cell lining on the insides of the lips, mouth, and throat (pharynx). An estimated 59,000 Americans will be newly diagnosed with oral cavity and oropharyngeal cancers in 2025 1, the majority of whom will undergo surgical resection as their primary treatment. A key challenge in the management of these patients is the spread of disease to regional lymph nodes in the neck, estimated to affect up to 50% of patients with primaries located in the oral cavity 2-4. Cancers in the oral tongue have the highest rate of cervical lymph node metastasis at the time of surgery compared to all other sites in the mouth 1,5. Unsurprisingly, neck disease is an important prognostic indicator for oral cancer patients, associated with higher rates of locoregional recurrence and poorer overall survival 6-8.

The detection of lymph node metastases in the neck presently relies on multimodal approaches combining manual palpation and anatomical imaging modalities like computed tomography (CT), magnetic resonance (MR), and ultrasound that use node morphology (e.g., volume) to identify suspicious nodes 9. The performance of these image-based approaches for detecting neck disease varies widely across studies depending on the patient populations analysed (e.g., early *vs* advanced stage, primaries of oral cavity *vs* oropharynx) and morphological criteria employed 10,11. Crucially, when studies compared the findings of neck examinations (palpation and imaging) to the pathological stage of dissected lymph nodes, between 10~30% of oral cancer patients with clinically negative (cN0) necks were revealed to have pathologically confirmed (pN+) lymph node metastases 12,13. Therefore, uncertainties over the neck status of patients from clinical examination have resulted in current recommendations for routine elective neck dissections in patients with high-risk primaries 9.

Molecular imaging techniques using radiotracers and optical probes have been increasingly explored for providing complementary information to CT and MR imaging for guiding the management of the necks of oral cancer patients 14. For example, the uptake of [^18^F]Fluorodeoxyglucose (^18^F-FDG) radiotracers in neck lymph nodes using positron emission tomographic (PET) imaging has demonstrated modest improvements for catching occult metastases in cN0 necks 11,15; yet ^18^F-FDG PET, like all the other imaging modalities, is insufficiently reliable for detecting microscopic neck disease to eliminate the need for elective neck dissections in high-risk patients 16-18. In intraoperative settings, real-time fluorescence (FL) imaging with optical probes targeting the tumour biology of oral cancers has shown promise in clinical studies for clearing surgical margins 19,20, and mapping metastatic and sentinel lymph nodes in the neck 21,22. Ultimately, there is still a need for imaging agents that can provide more accurate staging of neck disease preoperatively, guide lymph node dissections intraoperatively, and ideally both.

Our group has previously reported the development of Porphysomes (PS), multifunctional porphyrin-lipid nanotheranostics for fluorescence-guided surgery and photodynamic ablation of oral cancers 23-25. In a rabbit VX-2 buccal tumour model, Muhanna *et al.* demonstrated with FL imaging a > 2-fold enhancement of PS fluorescence in suspicious neck nodes, which was used to guide lymph node biopsies and neck dissections 23. The porphyrin building blocks of PS also serve as chelators for transition radiometals such as positron-emitting Copper-64 (^64^Cu) 26,27. Using ^64^Cu-radiolabelled PS, the authors showed that uptake of ^64^Cu-PS radiotracers in VX-2 oral tumours provided clear tumour delineation and detailed mapping of sentinel and metastatic lymph nodes in the rabbit neck on PET 23. Altogether these previous data suggest that multimodal ^64^Cu PET and FL imaging with PS may fill a major gap in the clinical management of occult neck metastases in oral cancer patients.

**Figure 1:**
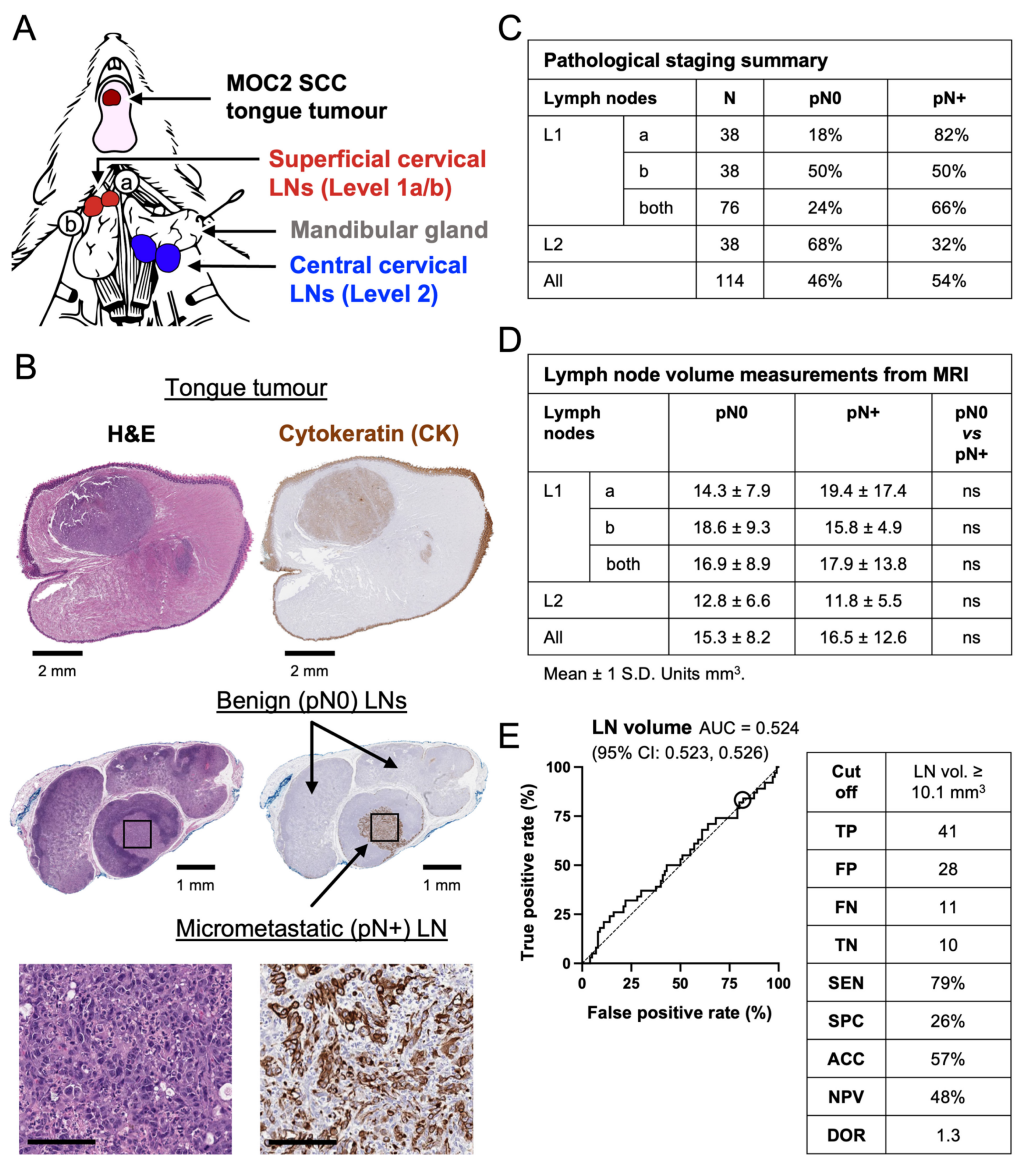
** Characterisation of the preclinical model of oral cancer with neck metastases.** (A) Head and neck anatomy of rodent model with a MOC2 tongue tumour. (B) Histopathology of tongue tumour and neck nodes containing a CK^+^ micrometastasis. Scale bar for histology insets 100 µm. (C) Summary of anatomical distribution and pathological staging of all neck nodes included in study. (D) Summary of lymph node volumes from MR imaging according to anatomical level and pathological staging. Mean ± 1 S.D. Units mm^3^. Statistics compare pN0 *vs* pN+ staged nodal volumes using multiple comparison t-tests with Tukey correction. (E) ROC curve and diagnostic performance of test predicting pN+ staged nodes using nodal volume from MR imaging. ROC AUC and 95% C.I.s listed in brackets.

The present study is a comprehensive investigation of ^64^Cu-labelled PS nanotheranostics for multimodal staging of metastatic disease in the cervical lymph nodes of preclinical models of oral cancer. A xenograft model of squamous cell carcinoma (SCC) was established in the tongues of nude rats, giving rise to reproducible micrometastases in the neck lymph nodes (Fig. 1). ^64^Cu-PS were administered either intratumourally (IT) or intravenously (IV), and their uptake in tongue tumours and cervical nodes imaged with serial PET/MR and *in situ* FL. After imaging, all the neck nodes were dissected and submitted for blinded pathological staging. Receiver operating characteristics (ROC) testing the accuracy for identifying pN0 *vs* pN+ staged lymph nodes were calculated for both ^64^Cu-PS PET and FL imaging modalities and compared to tests using nodal morphology (from MRI) or ^18^F-FDG uptake. Altogether, these studies aim to answer whether ^64^Cu-PS nanotheranostics permit more accurate multimodal detection of microscopic neck disease in preclinical oral cancer models *vs* existing clinical imaging modalities.

## Materials and Methods

### Materials and reagents

PS are PEGylated pyro-lipid nanoparticles 28 consisting of 55 mol% pyro-lipid (University Health Network, Toronto, Canada), 40 mol% cholesterol (CH-0355; CordenPharma, Liestal, Switzerland), and 5 mol% N-(carbonyl-methoxypolyethyleneglycol-2000)-1,2-distearoyl-sn-glycero-3-phosphoethanolamine (LP-R4-039; CordenPharma). ^64^Cu chloride ([^64^Cu]CuCl_2_) was purchased from the University of Wisconsin Institutes for Medical Research Cyclotron Labs (Madison, USA). Protocol for radiolabelling ^64^Cu-PS has been previously described 27: the radiochemical purity of ^64^Cu-PS used for animal experiments was 96.6 ± 1.1% from instant thin layer chromatography and the A_s_ was 503 ± 98 MBq ^64^Cu/mg pyro-lipid. Physicochemical properties of PS and ^64^Cu-PS are provided in Fig. S1. Chelation stability of ^64^Cu-PS has been previously confirmed under physiological conditions 26,27.^ 18^F-FDG was purchased from Isologic Innovative Radiopharmaceuticals (Toronto, Canada) and was used as supplied.

### Development of tongue tumour xenograft model with neck metastasis

Mouse oral squamous cell carcinoma (MOC2) cells (EWL00w-FP; Kerafast, Shirley, USA) derived from a chemokine receptor CXCR3-deficient C57BL/6 mouse 29 were cultured per the supplier’s protocol. Authentication testing of MOC2 cells was performed using short tandem repeat profiling and Mycoplasma testing completed by polymerase chain reaction. Cells were used for animal experiments after ≤ 10 passages. Female athymic nude rats (Hsd:RH-*Foxn1^rnu^*) were purchased from Invotiv (Livermore, USA). On the day of tumour implantation, animals weighed 221 ± 35 g. Under inhaled anaesthesia, a single cell suspension of 100,000 MOC2 cells in 0.15 mL phosphate buffered saline was injected into the body of the tongue using a 29 Ga needle. Post procedure, animals were housed in pairs, provided an irradiated alfalfa-free diet (TD.94045; Inotiv) and water *ad libitum*. Animal weight and clinical condition were monitored daily. Summary of experimental details for each animal is provided in Table S1.

### PET/MR imaging in tongue tumour model

PET/MR imaging was performed using a 1T PET/MR small animal scanner (nanoScan; Mediso, Budapest, Hungary). For intratumoural lymphatic mapping experiments, ^64^Cu-PS (80–120 MBq ^64^Cu, 0.5 mg pyro-lipid, IT) were administered into the tongue tumour in a 0.05–0.1 mL volume using a 31 Ga hypodermic needle. PET/MR imaging was performed at timepoints: 1 h, 3 h, 6 h, 12 h, 24 h, 48 h, and 72 h post-IT injection. PET acquisition times ranged from 10–25 min and T1 3D material maps were collected for MRI-based attenuation correction using parameters: 256 x 182 matrix, 0.35 x 0.35 x 0.60 mm^3^ voxel size, 18.98 ms repetition time, 2.9 ms echo time, 15° flip angle.

For comparison experiments between systemically injected PET radiotracers, ^18^F-FDG was injected and imaged 24 h prior to experiments involving ^64^Cu-PS. Rats were fasted overnight and blood glucose measured ~4.9 mmol/L before injection. ^18^F-FDG (46 MBq ^18^F/kg, IV) was administered via the tail vein and PET/MR imaging performed after ~45 min of uptake. PET acquisition time was 10 min using the same imaging parameters described above. The following day, ^64^Cu-PS (250–500 MBq ^64^Cu/kg, 0.5–1.0 mg/kg pyro-lipid, IV) were administered via tail vein and PET/MR imaging repeated as described above at 3 h and 24 h post-IV injection using a 15–20 min PET acquisition time.

All PET images were reconstructed using parameters: 400-600 keV energy window, 105 x 105 x 237 matrix, 0.4 mm^3^ voxel size. Image analysis was performed in Inveon Research Workplace (Ver. 4.2.0.15; Siemens Medical Solutions, Malvern, USA). Volumes of interest (VOIs) were manually created by trained readers for the tongue tumour, benign tongue, and any cervical lymph nodes visualised with PET and/or MR. The mean and maximum standardised uptake values (SUV) were generated for the tumour and lymph node VOIs for quantitative analysis and statistical testing. Lymph nodes that were nonvisible on PET imaging were given SUV values equivalent to background uptake in benign tongue. MIPs of PET/MR images were exported with arbitrary scales for PET intensity.

### *In situ* fluorescence imaging

Animals were euthanised and the cervical lymph nodes exposed. A white light image of the neck was captured. Animals were positioned in an IVIS Spectrum *In Vivo* Imaging System (PerkinElmer; Shelton, USA) for FL imaging using filter setting: 675 nm Ex, 720+ nm Em, 1–5 s exposure, 1.5 cm (height) focus. The FL images were opened in Live Image software (Ver. 5.4.2.18425; PerkinElmer) and regions of interest (ROIs) created over the nodes and mandibular gland. The mean and maximum fluorescence radiant efficiency (units = [p/s/cm^2^/sr]/[µW/cm^2^]) was extracted from each ROI. The relative fluorescence in the lymph nodes was calculated as signal-to-background (S/B) ratio using the ‘background’ fluorescence in the mandibular gland. The mean and maximum S/B ratios were calculated in the lymph node ROIs for quantitative analysis and statistical testing.

### Lymph node histopathology

The tongue tumours and lymph nodes were harvested for histological processing after imaging. Tissue dyes were used for marking the anatomical positioning of the neck nodes prior to submission. For routine processing, tissues were formalin fixed, paraffin embedded, and cut sections were stained for H&E and cytoplasmic cytokeratin (CK) (NBP2-29429; Novus Biologicals, Toronto, Canada) per laboratory standard procedures. Two or three levels, separated by ~0.25 mm, were cut from each paraffin block and slides scanned with 20x magnification. For ^64^Cu tissue autoradiography, the harvested tissues were embedded in O.C.T. compound (SGN4585; Fisher Healthcare, Houston, USA) and flash frozen in liquid nitrogen before cutting on a cryostat with 30 µm slice thickness. The autoradiography slides were exposed against a high-resolution storage phosphor screen (7001487; PerkinElmer) for ~24 h before imaging at 600 DPI (~42 μm/pixel) resolution using a phosphor imaging system (Cyclone Plus; PerkinElmer). Additional slides were cut from frozen blocks for H&E, CK, and CD68/SR-D1 (NB100-683; Novus Biologicals) staining and fluorescence microscopy using 4′,6-diamidino-2-phenylindole (DAPI) (D1306; Thermo Fisher Scientific) and FITC-tagged CK (NBP2-33200F; Novus Biologicals). Fluorescence scans (Axioscan 7; Carl Zeiss Microscopy, Jena, Germany) were acquired with 20x resolution using DAPI and Cy5 filters (Cy5 overlaps with the fluorescence wavelength of pyro-lipid).

Pathological staging of lymph nodes was performed by clinical readers blinded to the imaging data using the AJCC TNM cancer staging system 30,31: nodes with no detectable CK^+^ cellular staining or having isolated tumour cells defined as CK^+^ metastatic foci ≤ 0.2 mm and lacking clustering pattern were considered node-negative (pN0); nodes with micrometastasis defined as CK^+^ metastatic foci > 0.2 mm and ≤ 2.0 mm (or ~200 CK^+^ cell clusters) were considered node-positive (pN+). Slide scans were viewed and measured in QuPath (Ver. 0.5.1; The University of Edinburgh, Edinburgh, UK). No nodes in this study (N=114) presented with tumour deposits > 2.0 mm (macrometastasis). Extracapsular disease spread from lymph nodes was extremely rare (N=2).

### Receiver operating characteristics analysis

ROC curves were created by plotting true positive rate *vs* false positive rate. 95% C.I. for area under the ROC curve (AUC) was calculated using nonparametric methods. The optimal cutoff values of the diagnostic tests reported in the text and figures used the Youden index; results with other cutoff value methods are reported in Table S10. Equations for calculating diagnostic test results are provided in the Supplemental Data.

### Statistical analysis

Statistical testing was performed using Prism (Ver. 10.3.1 (464); GraphPad Software, Boston, USA) or MATLAB (Ver. R2023b Update 4; The MathWorks, Natick, USA). Post-hoc power analyses were performed using G*Power software (Ver. 3.1.9.6; Heinrich Heine Universität Düsseldorf, Düsseldorf, Germany) and are provided in the Supplemental Data. Mean ± 1 S.D. reported in text. Box and whisker plots use Tukey’s method and “+” to denote sample means. Multiplicity adjusted P values and ⍺ = 0.05 were used throughout. P values: not significant (ns) > 0.05, * ≤ 0.05, ** ≤ 0.01, *** ≤ 0.001.

## Results

### Development of an oral cancer neck metastasis model

By 10–14 d post-implantation of murine MOC2 SCC cells, the tongue tumours had reached 171 ± 84 mm^3^ from MR imaging and rat body weights had decreased ~5%. At the time of experimentation, 54% (61/114) of all the cervical lymph nodes harvested for histopathology demonstrated evidence of microscopic tumour cell clusters measuring > 0.2 mm from CK immunohistochemical staining (Fig. 1C). An additional 43 nodes had microscopic evidence of isolated CK^+^ tumour cells on at least one slide level; these nodes were staged pN0 in this study for consistency with clinical standards regarding isolated tumour cells 30,31. Neck nodes from anatomical level 1 (i.e., superficial cervical nodes) located closest to the tongue tumour exhibited a 66% (50/76) prevalence rate for pN+ staging whereas the more distantly located central cervical nodes in level 2 had a pN+ prevalence rate of 32% (12/38). There were no incidences of lymph node macrometastasis > 2 mm, nor any skip nodal metastases to the central (level 2) cervical nodes detected in this model. Overall, the pattern of cervical node metastases in our preclinical model is similar to the reported distribution pattern of neck metastases in clinical patients with primaries in the oral cavity 4,5.

The volumes of cervical lymph nodes were also measured with MR imaging and analysed retrospectively once the pathological stage was known. In Fig. 1D, no meaningful differences in the volumes of pN0 (15.3 ± 8.2 mm^3^) *vs* pN+ (16.5 ± 12.6 mm^3^) staged nodes were observed. A diagnostic test using lymph node volume (Fig. 1E) to identify nodal disease performed no better than a random classifier (i.e., coin flip) in this preclinical neck metastasis model.

### Sentinel and metastatic lymph node mapping with intratumoural ^64^Cu-PS injection was nondiagnostic

**Figure 2:**
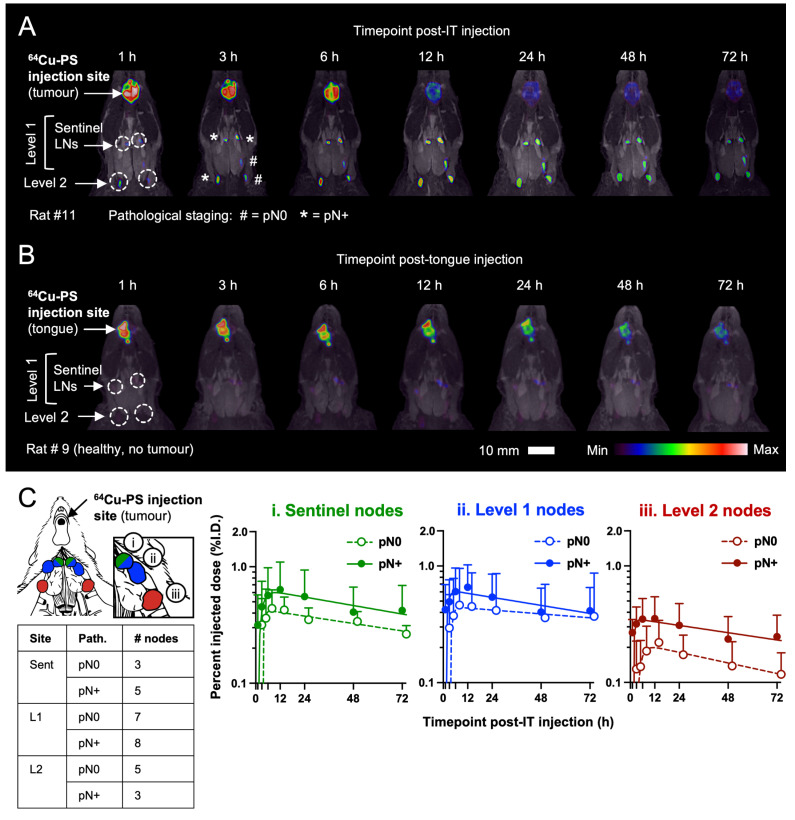
Lymphatic mapping in tongue tumour model with IT injection of ^64^Cu-PS. (A) Representative MIPs from serial ^64^Cu-PS PET/MR imaging post-IT injection (80–120 MBq ^64^Cu, 0.5 mg pyro-lipid). Pathological staging of nodes indicated on 3 h image. ^64^Cu PET signal intensity arbitrarily scaled to the 1 h timepoint. (B) Healthy rats (i.e., without tongue tumours) administered ^64^Cu-PS into the tongue and serially imaged. ^64^Cu PET signal intensity scaling as in (A). (C) Image-based analysis of ^64^Cu-PS pharmacokinetics in the sentinel lymph nodes, and in nodes from levels 1 and 2 by pathological status. Number of nodes listed in table for panel (C). Units %I.D. Means + 1 S.D. Trend lines are empirical fitting through mean value at each timepoint.

Lymphatic mapping with ^64^Cu-PS was performed in the tongue tumour model using serial PET/MR imaging to track the drainage of nanoparticles from the intratumoural injection site to the cervical lymph nodes (Fig. 2A). Lymph nodes were clearly visible in the neck from PET imaging starting as early as 1 h post-IT injection and reached a peak contrast by 12~24 h. Minimal cervical lymphatic drainage of ^64^Cu-PS nanotheranostics was observed after injection into the tongues of healthy rats (Fig. 2B), suggesting that lymphatic flow rate or volume draining from the tongue is enhanced by the presence of the tumour.

Analysing the pharmacokinetics of ^64^Cu-PS in the tumour-draining lymph nodes with respect to pathological staging (Fig. 2C) revealed that pN+ staged nodes exhibited both a greater absolute accumulation of nanoparticle and a longer retention time (i.e., larger AUC) post-IT injection. However, the kinetic differences between pN0 and pN+ neck nodes were not statistically significant at any level analysed (see Table S3). Considerable differences were noted in the clearance rates of ^64^Cu-PS from the IT injection site (t_1/2_ ~19.8 h) *vs* from the sentinel nodes (t_1/2_ ~89.7 h), suggesting potentially distinct biological mechanisms of nanoparticle retention in tumours and draining lymph nodes. In summary, ^64^Cu-PS PET signal in tumour-draining neck nodes following IT injection may be helpful for preoperative planning of neck dissections but does not provide reliable diagnostic information on the pathological staging of occult lymph nodes from oral cancers.

### ^64^Cu-PS passively accumulate in the tongue tumour following IV injection

**Figure 3:**
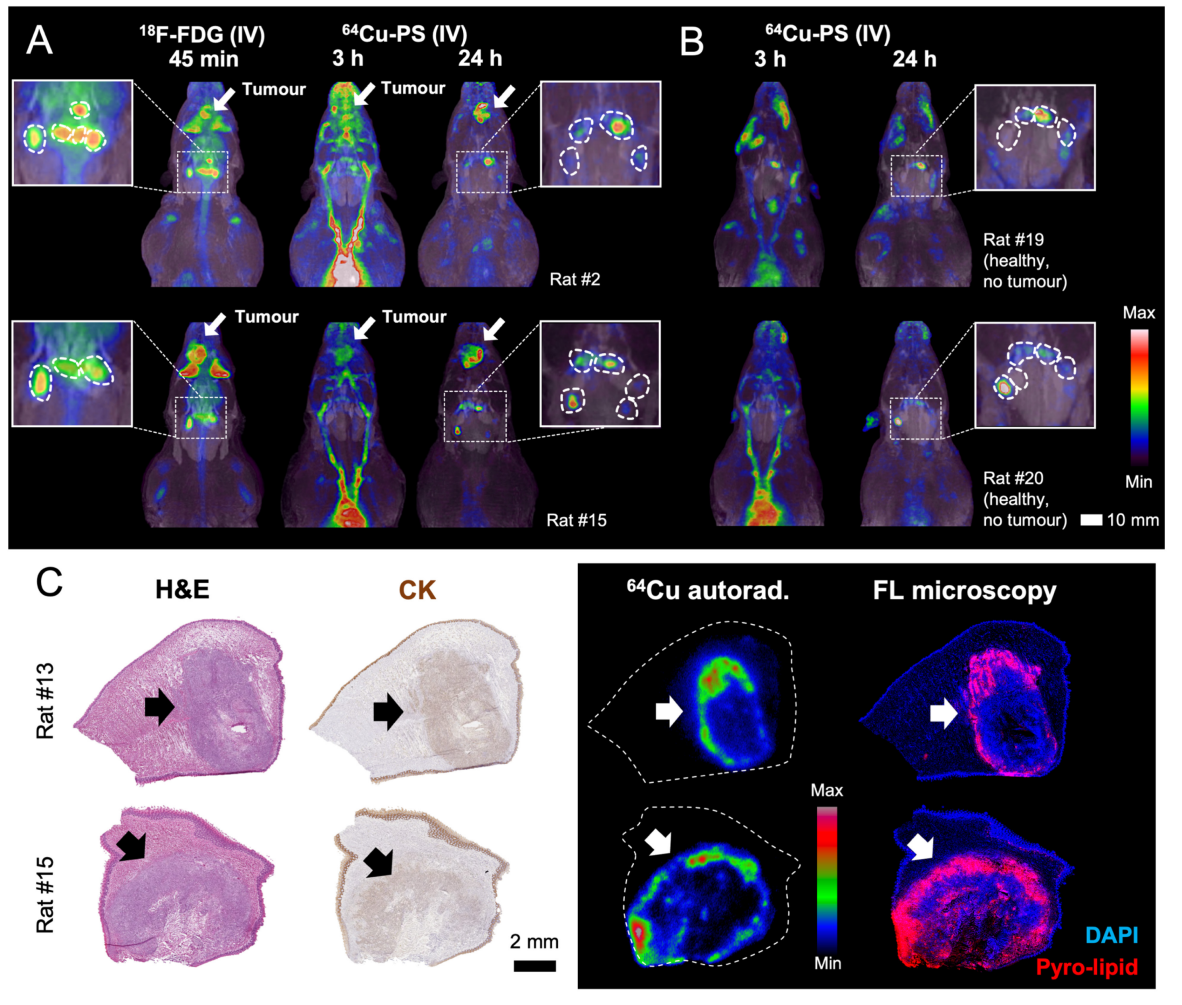
Uptake of ^64^Cu-labelled PS in tongue tumours. (A) Representative MIPs of ^18^F-FDG PET/MR images 45 min post-IV injection (46 MBq ^18^F/kg), and ^64^Cu-PS PET/MR images from 3 h and 24 h post-IV injection (250–500 MBq ^64^Cu/kg, 0.5–1.0 mg/kg pyro-lipid) in tumour-bearing rats. Magnified view of outlined neck nodes in insets. Arrows denote tumour. ^64^Cu PET signal intensity arbitrarily scaled to the 3 h timepoint. (B) Representative MIPs of ^64^Cu-PS PET/MR images from healthy rats (i.e., without tongue tumours). (C) Representative histopathology of rat tongues with SCC tumours illustrating correspondence between CK^+^ stained tumour cells, ^64^Cu autoradiography signal, and pyro-lipid FL microscopy. Arrows denote tumour.

In the context of oral cancers, IV injection is a more useful route for administering imaging agents owing to the highly complex patterns of lymphatic drainage in the neck (and correspondingly complex pathways of possible lymphatic metastasis from oral cavity tumours) 32,33. The kinetics of ^64^Cu-PS uptake in the preclinical oral cancer model was first established using serial PET/MR imaging at 3 h and 24 h post-IV injection (Fig. 3). PET imaging using ^18^F-FDG (^18^F physical t_1/2_ 109 min) was also performed in the same rats 24 h prior to ^64^Cu-PS experiments to compare differences in uptake between the canonical glucose metabolism-based PET radiotracer and nontargeted PS nanoparticles.

After IV injection, ^64^Cu-PS were predominantly in blood circulation at the 3 h imaging timepoint and significant nonspecific PET signal was observed from healthy tissues in the neck and mouth (Fig. 3A). By 24 h, far better PET image contrast was obtained in the tongue tumour and the cervical lymph nodes, similar to the contrast seen with ^18^F-FDG PET after 45 min uptake. From 3 h to 24 h post-IV injection, the SUV_mean_ of ^64^Cu-PS nanoparticles in the tongue tumour significantly increased from 2.41 ± 0.73 to 4.39 ± 1.80 (P value < 0.001, Power = 0.83, see Fig. S2). In healthy rats without tongue tumours, low and nonspecific uptake of ^64^Cu-PS was observed in the cervical nodes (Fig. 3B), similar to the pattern observed in the necks of healthy rats after local injection of nanoparticles into the tongue (see Fig. 2B).

The localisation of ^64^Cu-PS in CK^+^ squamous cell tumours was visualised in tongue histology using tissue autoradiography and fluorescence microscopy (Fig. 3C). A high correspondence between the ^64^Cu autoradiography intensity and the fluorescence signal from the pyro-lipid building blocks of PS was observed, suggesting excellent chelation stability of ^64^Cu-PS *in vivo*. Nonuniform distribution of ^64^Cu-PS in the tumour cross-section was also noted, favouring the tumour periphery where it is presumed that blood vessels are still well perfused and the draining lymph vessels are functionally patent.

### ^64^Cu-PS PET detects metastatic lymph nodes with greater accuracy than ^18^F-FDG PET

**Figure 4:**
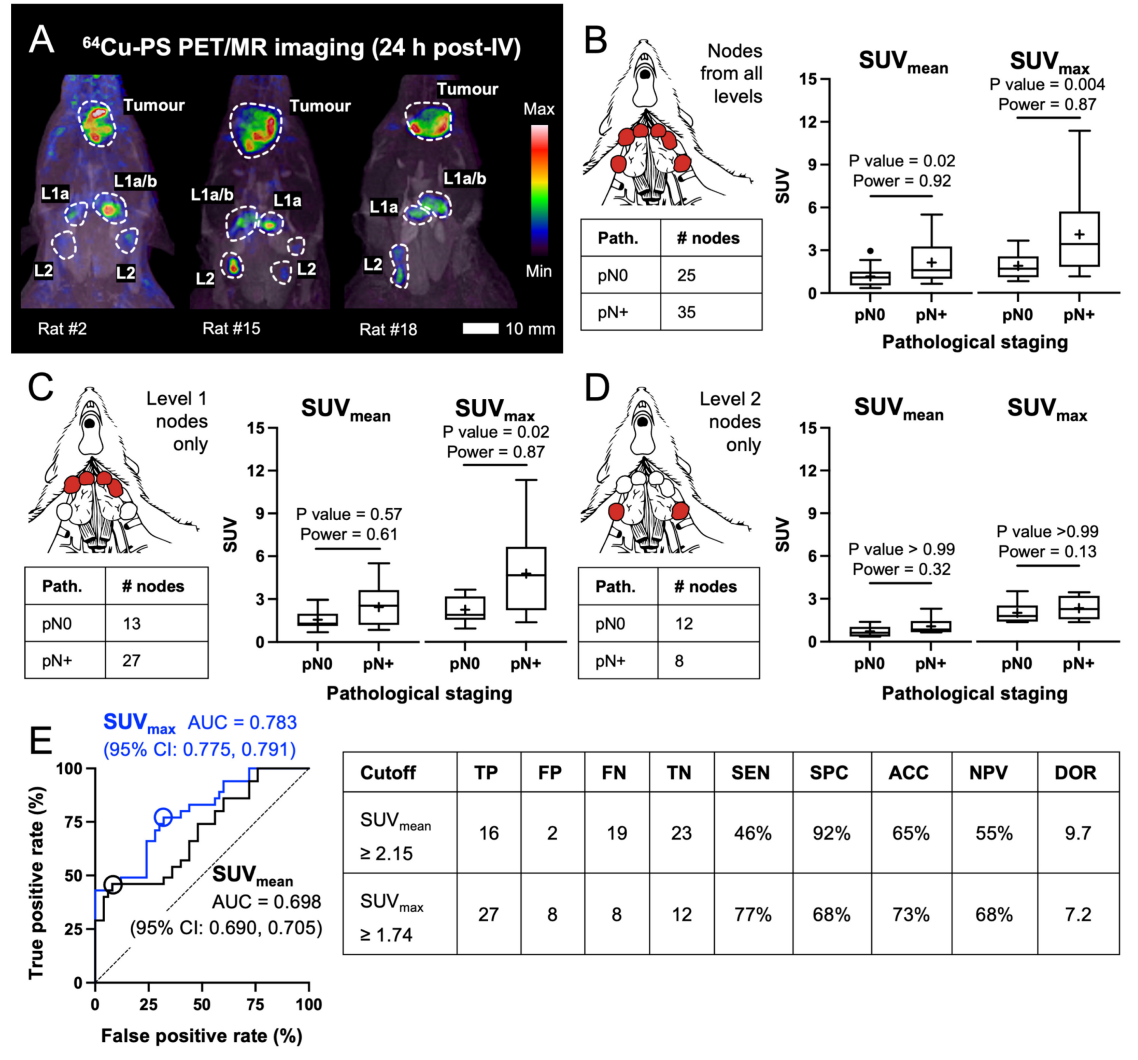
PET imaging of ^64^Cu-labelled PS uptake in neck lymph nodes of tongue tumour models. (A) Representative MIPs of ^64^Cu-PS PET/MR images from 24 h post-IV injection (250–500 MBq ^64^Cu/kg, 0.5–1.0 mg/kg pyro-lipid). ^64^Cu-PS uptake in pN0 *vs* pN+ staged nodes from (B) all anatomical levels, (C) level 1 only, and (D) level 2 only. Tukey box-and-whisker plots with “+” at mean. SUVs unitless. Statistics compare pN0 *vs* pN+ staged nodes using multiple comparisons t tests with Tukey correction. Power analysis is a post hoc test of two independent means. (E) ROC curves and diagnostic performance of tests predicting pN+ staged nodes using 24 h PET imaging SUV. ROC AUCs and 95% CIs listed in brackets.

As observed in Fig. 3A, the PET imaging contrast from ^64^Cu-PS in the tongue tumour and cervical lymph nodes was optimal at 24 h post-IV injection (Fig. 4A). Nearly all the neck nodes (50/60, or 83%) harvested for histopathology were successfully visualised with ^64^Cu-PS PET imaging at 24 h, two more than were visualised with ^18^F-FDG PET. The neck nodes missed by ^64^Cu-PS PET imaging were exclusively from anatomical level 2 and only 2/10 (20%) missed nodes contained pathologically confirmed micrometastases.

Pooling together the uptake in lymph nodes from all anatomical levels of the neck, there was significantly greater uptake in pN+ *vs* pN0 staged nodes measured using both SUV_mean_ and SUV_max_ (Fig. 4B). Analysing neck nodes from anatomical level 1 only, the difference in SUV_max_ between pN0 and pN+ nodes was also statistically significant with sufficient power (Fig. 4C). In level 2, pN+ staged nodes had slightly higher uptake than pN0 nodes but this difference was not significant for either SUV_mean_ or SUV_max_ (Fig. 4D). From the uptake data of ^18^F-FDG PET imaging in the same rats, we observed no significant difference in SUV between pN0 and pN+ staged nodes and in fact greater uptake of ^18^F-FDG on average in the pN0 nodes (see Fig. S3).

From the ROC curves for ^64^Cu-PS PET imaging in Fig. 4E, a greater AUC was obtained using SUV_max_ and completely overlapped the ROC curve for SUV_mean_. Using an optimal cutoff value of SUV_max_ ≥ 1.74 provided 77% SEN, 68% SPC, 73% ACC, and 68% NPV for diagnosing pN+ staged nodes. False positive nodes were mostly from anatomical level 1 (63%), while 5/8 (63%) false negative nodes from ^64^Cu-PS PET were from level 2. In contrast to ^64^Cu-PS, using the SUV_max_ from ^18^F-FDG PET imaging achieved only 81% SEN, 43% SPC, 67% ACC, and 59% NPV for diagnosing pN+ staged nodes (see Fig. S3).

### *In situ* ^64^Cu-PS FL imaging outperformed PET imaging for detection of neck disease

**Figure 5:**
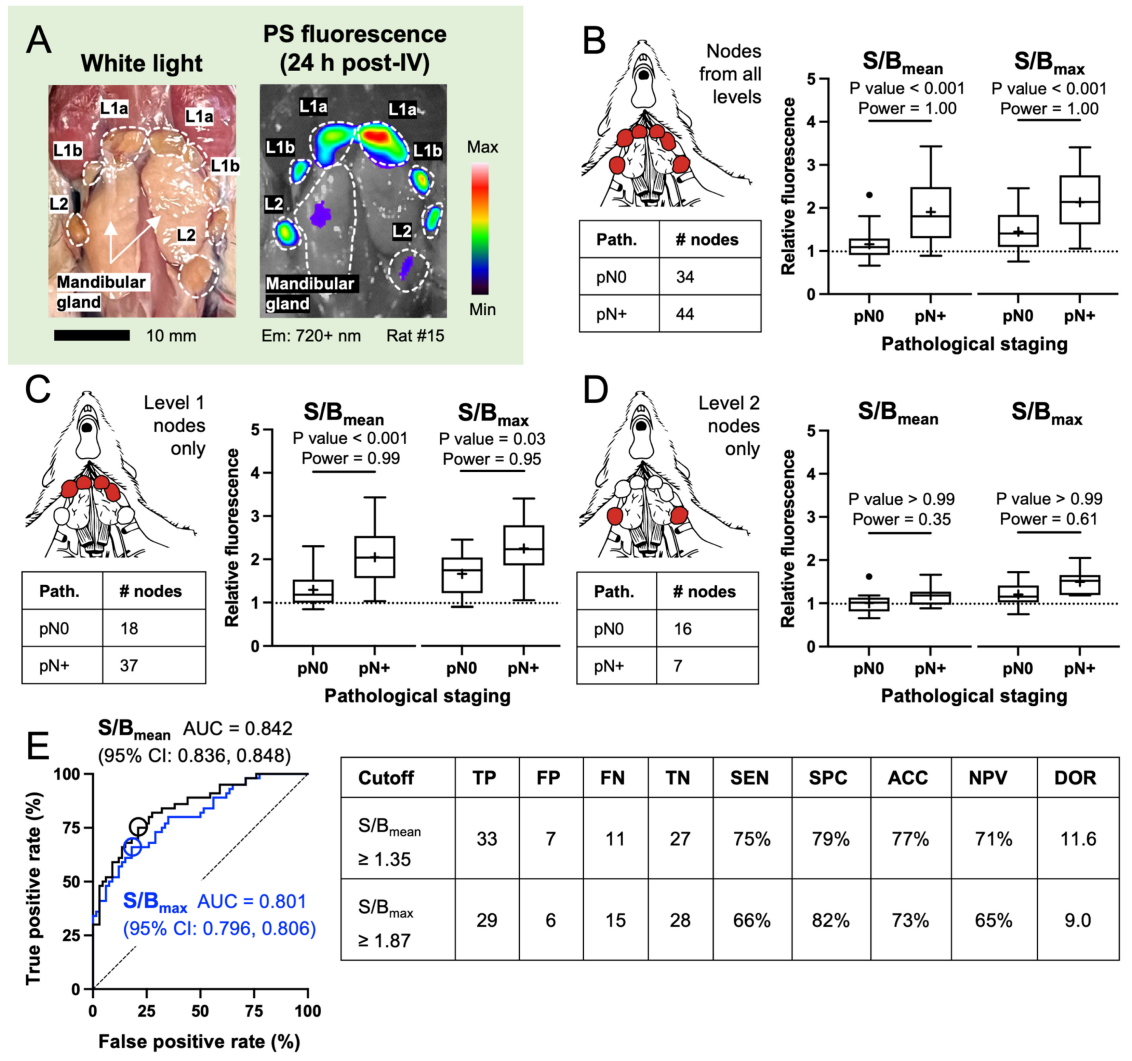
*In situ* FL imaging of ^64^Cu-labelled PS fluorescence in neck lymph nodes of tongue tumour models. (A) Representative white light and PS fluorescence images of neck nodes 24 h post-IV injection (0.5–1.0 mg/kg pyro-lipid). Relative PS fluorescence in pN0 *vs* pN+ staged nodes from (B) all anatomical levels, (C) level 1 only, and (D) level 2 only. Tukey box-and-whisker plots with “+” at mean. S/B ratios unitless. Statistics compare pN0 *vs* pN+ staged nodes using multiple comparisons t tests with Tukey correction. Power analysis is a post hoc test of two independent means. (E) ROC curves and diagnostic performance of tests predicting pN+ staged nodes using FL imaging S/B. ROC AUCs and 95% CIs listed in brackets.

After PET imaging at 24 h post-IV injection, the rats were sacrificed and the neck nodes exposed for *in situ* FL imaging of ^64^Cu-PS (Fig. 5). Note that the PS fluorescence signal (emission peak at 671 nm) was not strong enough to visualise through the skin. In Fig. 5A, the lymph nodes were clearly visualised amidst the complex anatomy of the neck using PS fluorescence: 86% (67/78) of all the cervical lymph nodes harvested for histopathology were more fluorescent than the mandibular gland (selected as background for the S/B calculations).

Pooling together the S/B ratios of nodes from all anatomical levels in the neck, significantly greater relative PS fluorescence was detected in pN+ *vs* pN0 staged nodes whether using S/B_mean_ or S/B_max_ ratios (Fig. 5B). Analysing the nodes from level 1 only, we observed that the difference in PS fluorescence between pN0 and pN+ nodes was still statistically significant and sufficiently powered for both ratios (Fig. 5C). However, pN+ staged nodes from level 2 only were just slightly more fluorescent than pN0 nodes and from background fluorescence in the mandibular gland (Fig. 5D). Objectively, lymph nodes from level 2 were less fluorescent on average than those from level 1 receiving lymphatic drainage directly from the tongue tumour; this observation matches the data trend from ^64^Cu-PS PET imaging showing higher uptake in level 1 *vs* level 2 nodes at 24 h post-IV injection.

From the ROC curves for *in situ* ^64^Cu-PS FL imaging in Fig. 5E, the greatest AUC was obtained using the S/B_mean_ ratio for diagnostic testing. The curve for S/B_mean_ also overlapped the ROC curves for S/B_max_ and from ^64^Cu-PS PET imaging in Fig. 4E, suggesting that ^64^Cu-PS FL imaging can be used as a standalone modality for diagnosing lymph node metastasis in this preclinical oral cancer model. Using an optimal cutoff value of S/B_mean_ ≥ 1.35 provided 75% SEN, 79% SPC, 77% ACC, and 71% NPV for pN+ staged nodes. Nearly all false positive nodes were from anatomical level 1 (86%) and completely overlapped with the false positive nodes from ^64^Cu-PS PET. Nodes falsely labelled negative from FL imaging came evenly from both anatomical levels 1 (45%) and 2 (55%) and included the nodes mislabelled negative from ^64^Cu-PS PET.

## Discussion

The therapeutic benefits from elective neck dissections in early-stage oral cancer patients with cN0 necks are well evidenced 12,34, yet come at the expense of several treatment-related functional and cosmetic morbidities and impacted quality of life 35,36. The motivation for these procedures is the estimated 10~30% of cN0 patients 12,13 whose undetected nodal disease creates significant risks for neck recurrences 37 requiring salvage treatments. Clinically occult neck metastases are typically microscopic, making their sensitive detection by preoperative imaging modalities difficult 18. This unmet clinical need has motivated the development of novel contrast agents like ^64^Cu-PS nanotheranostics to better detect occult neck metastases and potentially obviate the need for routine elective neck treatments altogether.

For this study, a T cell deficient athymic nude rat 38 was used to establish the oral cancer model with neck metastases (Fig. 1). Histological comparisons of cervical lymph nodes from athymic nude and immune competent rats have found microscopic differences in B cell zones like germinal centres (e.g., smaller and scarcer in nude rats), whereas T cell zones like the paracortex were still distinguishable, albeit laden with macrophages 39. Several preclinical head and neck cancer models have been described in immune competent rodents 40,41, but these typically lack reproducible development of cervical node metastases 42,43. In immune deficient animals, higher incidences of neck disease are obtained 42, possibly from reduced tumour-immune cell interactions combating the seeding of lymphatic metastases. The role of T cell interactions on nanoparticle trafficking to metastatic lymph nodes is underexplored 44,45 with literature pointing to dominant interactions of smaller, neutrally charged particles like ^64^Cu-PS with resident CD11b^+^ macrophages and follicular B220^+^ B cells, and only minor interactions with CD8^+^ T cells 46-50.

In oral cancers, known changes to CD20^+^ B cell and CD8^+^ T cell localisation in metastatic cervical lymph nodes 51 may enhance interactions with nanoparticles in a way that is favourable for their selective retention in metastatic nodes. Unravelling the roles of specific lymphocyte populations on the differential uptake of ^64^Cu-PS in benign and metastatic nodes (see Figs. 4, 5) was beyond this study’s scope and warrants further investigation in immune competent metastasis models (e.g., rabbit VX-2 oral tumours 23,24). Notably, such detailed cellular characterisations were not necessary for the successful clinical translation of fluorophore-labelled antibodies 52,53 and pH sensitive fluorescent micelles 54,55 for intraoperative FL imaging of oral SCC disease.

The clinical standard of care using elective neck dissections for early-stage cN0 disease is being challenged by less invasive, less morbid sentinel node biopsy 56,57; a technique measuring IT injected nanocolloidal radiotracer uptake in the first echelon cervical lymph node(s) with lymphoscintigraphy 58. In Fig. 2 we demonstrate that IT injection of ^64^Cu-PS similarly maps sentinel and other cervical nodes by PET imaging for up to 72 h. Uptake in pN+ staged nodes was higher on average than in pN0 nodes, likely reflecting greater lymphatic flow from the tongue tumour 59 and lower retention of particles in benign nodes 60. However, ^64^Cu-PS PET signal differences between pN0 *vs* pN+ staged nodes were not significant enough to be diagnostic outright (Table S3), as is also the case with existing sentinel node radiotracers. A potential advantage of ^64^Cu-PS is their inherent multimodality imaging, combining high resolution preoperative PET and intraoperative FL imaging. Several of the pitfalls with the sentinel lymph node biopsy in oral cancers—obstructed or redirected lymphatic flow 61, skip nodal metastases 62, etc.—will similarly affect the use of IT-injected ^64^Cu-PS and motivate exploring IV administration.

Following systemic injection, ^64^Cu-PS passively accumulated in the tongue tumour and drained to the cervical lymph nodes, yielding high contrast PET images of the tongue and neck by 24 h post-IV injection comparable to ^18^F-FDG PET (Fig. 3A). Contrast from FL signal in lymph nodes *vs* background was also exceptionally high (Fig. 5A). ^64^Cu-PS signal was measurably greater on average in pN+ *vs* pN0 staged lymph nodes by both PET (Fig. 4B-D) and *in situ* FL (Fig. 5B-D). The ROC AUC for ^64^Cu-PS PET SUV_max_ was 0.783 (Fig. 4E) which was significantly greater than the 0.575 AUC for ^18^F-FDG SUV_mean_ (Fig. S3). The highest ROC AUC obtained experimentally was 0.842 from ^64^Cu-PS FL S/B_mean_ (Fig. 5E) which compares favourably to other clinically tested imaging agents for oral SCC disease 63,64. The diagnostic performance of FL imaging S/B ratio superseded those with PET, confirming that *in situ* ^64^Cu-PS FL imaging can serve as a standalone intraoperative modality.

An influential paper by Weiss *et al.* 65 on the management of oral cancer patients with cN0 staged necks established that if the probability of cervical lymph node metastasis exceeds 20%, then treatment of the neck by surgery or radiation is warranted. Thus, a diagnostic test must achieve NPV > 80% (i.e., probability that a node is pN0 after a “negative” imaging result) to waive elective neck treatments. Using the Youden index no imaging modality studied here exceeded 80% NPV (Table S10). With an algorithm optimising cutoff values for NPV ≥ 80%, only ^64^Cu-PS imaging achieved this threshold: FL S/B_mean_ ratio ≥ 1.02 provided an 88% NPV with the trade-off of a 68% positive post-test probability from significant overstaging (i.e., type I error). Interestingly, across both imaging modalities lymph nodes from level 1 were consistently responsible for most “false positives” while missed “false negatives” originated primarily from level 2, a trend not seen with other agents like ^18^F-FDG 16 and fluorophore-labelled antibodies 63,66.

Diagnostic imaging of head and neck cancers often include comparisons of lymph nodes by neck level and/or by neck side (e.g., ipsilateral *vs* contralateral, depending on primary tumour location) to improve accuracy for regional metastasis detection. In this study ^64^Cu-PS signal differences between pN0 and pN+ nodes were analysed either pooled altogether (as in Figs. 4B, 5B) or separately by anatomical neck level (Figs. 4C-D, 5C-D). Neck level analyses were complicated by small sample sizes that underpowered statistical comparisons, particularly for level 2 nodes where the metastatic yield was only 11% (or 12/114 nodes, see Fig. 1C). At least 7 additional tumour-bearing rats would have been needed to sufficiently power this comparison, which given the modest differences between pN0 *vs* pN+ nodes at level 2 in ^64^Cu-PS uptake (Fig. 4D) and relative FL (Fig. 5D) did not seem worthwhile.

The biological mechanisms by which nontargeted ^64^Cu-PS preferentially accumulate in micrometastatic lymph nodes remain incompletely understood. Despite nonspecific uptake of IV injected ^64^Cu-PS in the cervical neck nodes of healthy rats without tumours (Fig. 3B), the uptake (Fig. S4) and relative FL (Fig. S5) in benign and malignant nodes of tumour-bearing rats was greater on average. This observation suggests that increased lymphatic tumour drainage 59, enabling greater free particle flow through ~100 µm wide lymph vessels, was the dominant delivery pathway for ^64^Cu-PS to the neck nodes. Vascular related mechanisms of accumulation like the enhanced permeability and retention (EPR) effect have been previously shown to occur only at more advanced stages of lymphatic disease, once metastatic deposits have evolved their own blood supply 60,67; thus, EPR effect was not likely to have been a leading pathway for particle uptake in lymph node micrometastases.

**Figure 6:**
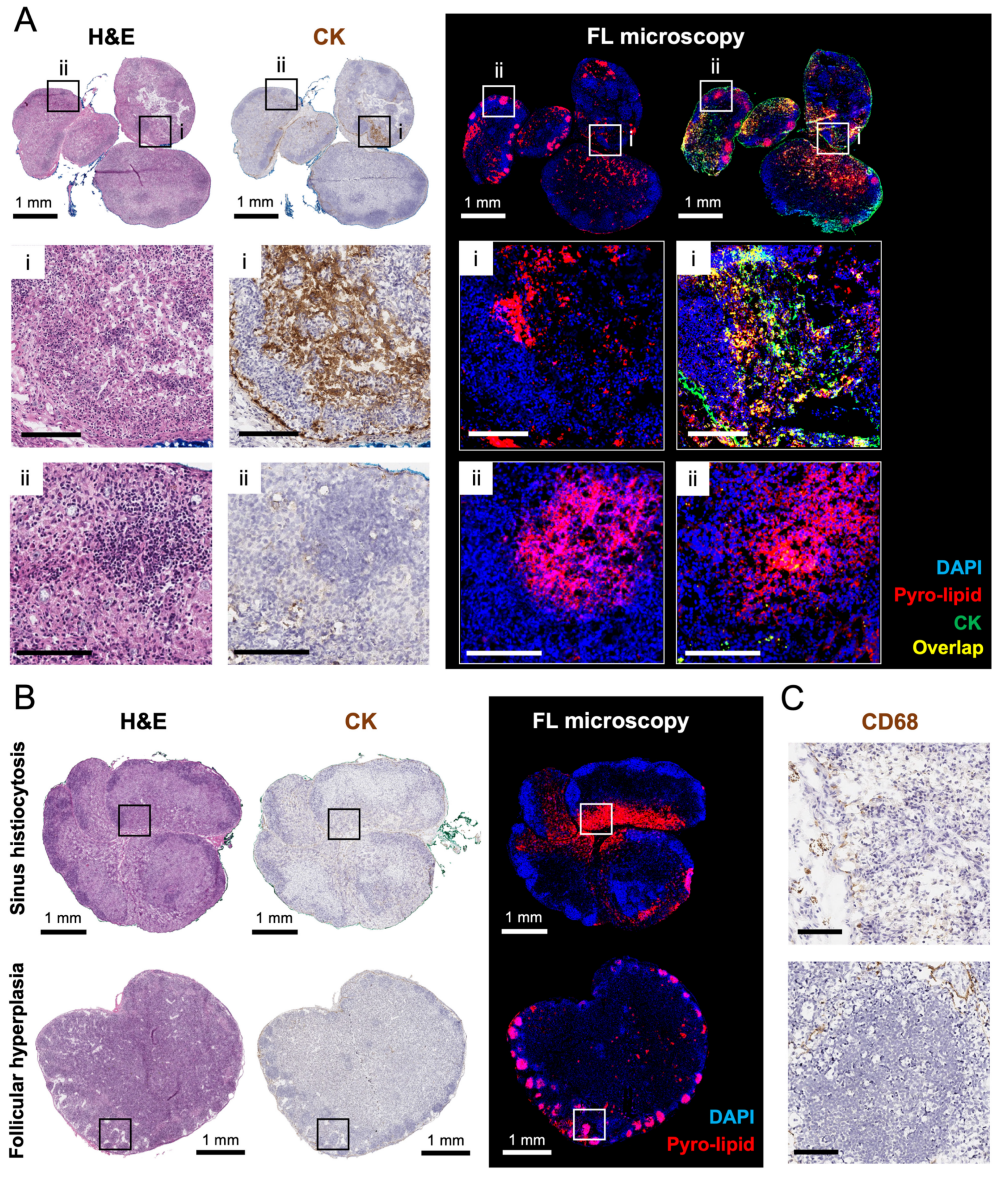
Histological microdistribution of^ 64^Cu-PS in neck lymph nodes of tongue tumour models. (A) Colocalisation of pyro-lipid FL signal with CK^+^ stained SCC tumour cells in a pN+ staged “true positive” lymph node from ^64^Cu-PS PET and FL imaging. Magnified views of pyro-lipid FL signal overlapping with (i) CK^+^ tumour cells in the subcapsular sinus and (ii) in tumour-free nodal structures such as B cell rich follicles. (B) Examples of pN0-staged “false positive” lymph nodes from ^64^Cu-PS PET and FL imaging illustrating nonspecific particle uptake in inflamed subcapsular sinuses (sinus histiocytosis) and follicles (follicular hyperplasia) with (C) CD68^+^ staining for lymph node-resident dendritic cells and macrophages. Scale bar for all histology insets 100 µm.

In Fig. 6A is histological evidence for the colocalisation of the fluorescent pyro-lipid building blocks with CK^+^ tumour cells in the subcapsular sinus, as well as evidence for nonspecific retention of particles in other benign structures of metastatic nodes such as lymphoid follicles. For nanoparticles with similar physicochemical properties to ^64^Cu-PS (i.e., ~80 nm diameter, neutral PEGylated surface), literature describes variable uptake in B220^+^ B cells microscopically located in the lymph node cortex 48,49 and in lymph node resident CD68^+^ dendritic cells and macrophages located in the subcapsular and medullar sinuses 46-48,50. The follicular uptake pattern of ^64^Cu-PS in Fig. 6A was consistent across both pN0 and pN+ staged nodes, an unexpected observation when compared to other nanoparticle uptake studies in benign or malignant lymph nodes 47,49,50,60. This uptake pattern may reflect underlying biological differences in the lymph nodes of immune compromised rodents 39, increased translocation of macrophages to B cell rich follicles after ^64^Cu-PS phagocytosis (i.e., for antigen presentation) 48,49, or even unintentional reprogramming of lymph node resident macrophages to pro-inflammatory M1 phenotypes from the low-level radioactivity of ^64^Cu 68, all hypotheses warranting further investigation.

Reactive and inflamed lymph nodes (lymphadenitis) frequently confound diagnostic tests for lymph node metastases in head and neck cancer, leading to “false positive” findings on ^18^F-FDG PET 69,70, with antibody-based optical imaging 63,66, and other nanoparticle-based theranostic imaging strategies 71,72. Two examples of pN0-staged “false positive” nodes from both ^64^Cu-PS PET and FL imaging are illustrated in Fig. 6B: significant particle uptake was observed in tumour-free lymph node structures with microscopic evidence of immunological reaction 73 and staining for CD68^+^ macrophages and dendritic cells (see Fig. 6C). Nonspecific ^64^Cu-PS uptake and retention in inflamed nodes may have prognostic relevance for patients, highlighting potential pre-metastatic niches in the neck for tumour cell colonisation 74 and sites of regional recurrences. Strategies for enabling clearer interpretation of benign, reactive, and metastatic lymph nodes, including serial imaging protocols 75,76 and dual-tracer injection approaches 77, have shown promise for other agents and may warrant further study with ^64^Cu-PS.

Facing the future clinical translation of PS nanotheranostics, our group has completed extensive studies of PS pharmacology and toxicology in rodents and beagle dogs 27. PS pharmacokinetics was dose proportional over a wide dose range, and single IV doses up to 32.6 mg/kg were safe in rats (compared to 0.5–1.0 mg/kg doses used here). A Good Manufacturing Practices-qualified radiopharmaceutical kit for labelling PS with 850 MBq [^64^Cu]CuCl_2_ was developed 27, and is producing patient doses for a first-in-human PET imaging study in metastatic cancer patients (NCT06977126). A 200 MBq ^64^Cu-PS dose was selected for patients based on prior clinical data with radiolabelled liposomes 78 and is predicted to give an effective radiation dose in patients between 7.6–9.4 mSv 27, or about half the radiation dose from a routine ^18^F-FDG oncology scan (14.1 mSv average effective dose 79). Future study phases will expand the eligibility for ^64^Cu-PS to include oral cancer patients with known or suspected regional lymph node involvement and scheduled for neck dissection.

## Conclusions

In summary we have demonstrated the diagnostic performance of ^64^Cu-PS nanotheranostics for identifying metastatic cervical lymph nodes in rats bearing tongue tumours. These nontargeted agents permitted more accurate detection of microscopic neck disease than conventional imaging techniques such as MR imaging (for cervical lymph node morphology) and molecular imaging using metabolic ^18^F-FDG PET. The unique multifunctionality of ^64^Cu-PS combining PET and near infrared FL imaging enables both pre- and intraoperative guidance for planning and performing neck dissections in oral cancer patients with greater precision. Altogether these results motivate the continued development of porphyrin-lipid nanotheranostics for clinical studies in oral cancer patients with clinically occult neck disease.

## Supplementary Material

Supplementary figures and tables.

## Data Availability

The data generated in this study are available upon request.

## Abbreviations

ACC: accuracy
A_s_: specific activity
AUC: area under the curve
CK: cytokeratin staining
C_max_: maximum tissue concentration
cN0: clinical lymph node-negative status
^64^Cu-PS: ^64^Cu-Porphysomes, PEGylated [^64^Cu]pyro-lipid nanoparticles
CT: computed tomographic imaging
DAPI: 4′,6-diamidino-2-phenylindole
DOR: diagnostic odds ratio
EPR: enhanced permeability and retention
^18^F-FDG: [^18^F]Fluorodeoxyglucose
FL: fluorescence imaging
FN: false negative
FP: false positive
H&E: haematoxylin and eosin staining
%I.D.: percent injected dose
IT: intratumoural
IV: intravenous
LN: lymph node
MIP: max intensity projection
MOC2: mouse oral squamous cell carcinoma
MR: magnetic resonance imaging
N/A: not applicable
NPV: negative predictive value
PET: positron emission tomographic imaging
pN0: pathological lymph node-negative status
pN+: pathological lymph node-positive status
PPV: positive predictive value
PS: Porphysomes, PEGylated pyro-lipid nanoparticles
ROC: receiver operating characteristic curve
ROI: region of interest
S/B: signal-to-background ratio
SCC: squamous cell carcinoma
SEN: sensitivity
SPC: specificity
SUV: standardised uptake value
TN: true negative
TP: true positive
VOI: volume of interest
