# Supplementary material for: Porphyrin-lipid nanotheranostics for multimodal imaging of nodal disease in preclinical oral cancers
Source: Theranostics. 2026 May 29;16(13):7108–23. doi: 10.7150/thno.129853 (PMC13294812; doi:10.7150/thno.129853)
Supplement: Supplementary file 1 — Supplementary figures and tables. [file thnov16p7108s1.pdf]

TITLE: Porphyrin-lipid nanotheranostics for multimodal imaging of nodal disease in preclinical oral cancers

AUTHORS: Michael S. Valic (ORCID: 0000-0003-3488-023X)[1,2], Esmat Najjar[1,3,4], Mark Zheng[1], Jason L. Townson[1,3], Harley H. L. Chan[1,3], Sharon Tzelnick[1,3,4], Alessandra Ruaro[1,3,4], Abdullah El-Sayes[1], Michael Halim[1], Pamela Schimmer[1], Chris J. Zhang[1], Tina Ye[1], Wenlei Jiang[1], Juan Chen[1], Jonathan C. Irish (ORCID: 0000-0002-1631-2717)[1,3,4], and Gang Zheng (ORCID: 0000-0002-0705-7398)[1,2,5]

AFFILIATIONS:

[1]Princess Margaret Cancer Centre, University Health Network, Toronto, Canada.

[2]Institute of Biomedical Engineering, Faculty of Applied Science and Engineering, University of Toronto, Toronto, Canada.

[3]Guided Therapeutics Program, University Health Network, Toronto, Canada.

[4]Department of Otolaryngology–Head and Neck Surgery, Temerty Faculty of Medicine, University of Toronto, Toronto, Canada.

[5]Department of Medical Biophysics, Temerty Faculty of Medicine, University of Toronto, Toronto, Canada.

CORRESPONDING AUTHORS:

Jonathan C. Irish, Princess Margaret Cancer Centre, University Health Network, 200 Elizabeth Street, 8NU-882, Toronto, ON M5G 2C4, Canada. E-mail: jonathan.irish@uhn.ca.

Gang Zheng, Princess Margaret Cancer Centre, University Health Network, 101 College Street, PMCRT RM 5-354, Toronto, ON M5G 1L7, Canada. Phone: 416-581-7667; E-mail: gang.zheng@uhn.ca.

Table of Contents

1. ABBREVIATIONS 3

2. DISCLAIMERS 4

3. SUPPLEMENTARY MATERIALS AND METHODS 5

3.1. Physicochemical nanoparticle characterisation *5*

3.2. Formula for ROC analysis *5*

4. SUPPLEMENTARY TABLES 7

Table S1. ..... *7*

Table S2. ..... *9*

Table S3. ..... *10*

Table S4. ..... *11*

Table S5. ..... *12*

Table S6. ..... *14*

Table S7. ..... *15*

Table S8. ..... *16*

Table S9. ..... *17*

Table S10. ..... *19*

5. SUPPLEMENTARY FIGURES 21

Figure S1. ..... *22*

Figure S2. ..... *23*

Figure S3. ..... *24*

Figure S4.. ..... *25*

Figure S5. ..... *27*

1. ABBREVIATIONS

ACC: accuracy; A_s: specific activity; AUC: area under the curve; $C_{max:}$ maximum tissue concentration; CI: confidence interval; ^{64}Cu-PS: ^{64}Cu-PORPHYSOMES, PEGylated [^{64}Cu]pyro-lipid nanoparticles; DLS: dynamic light scattering; DOR: diagnostic odds ratio; ^{18}F-FDG: [^{18}F]Fluorodeoxyglucose; FL: fluorescence imaging; FN: false negative; FNR: false negative rate; FP: false positive; FPR: false positive rate; %I.D.: percent injected dose; IT: intratumoural; IV: intravenous; LN: lymph node; MIP: mean intensity projection; MR: magnetic resonance imaging; N/A: not applicable; NPV: negative predictive value; PALS: phase analysis light scattering; PDI: polydispersity index; PET: positron emission tomographic imaging; pN0: pathological lymph node-negative status; pN+: pathological lymph node-positive status; PPV: positive predictive value; PS: PORPHYSOMES, PEGylated pyro-lipid nanoparticles; RCP: radiochemical purity; ROC: receiver operating characteristic curve; ROI: region of interest; S/B: signal-to-background ratio; SEN, sensitivity; SPC: specificity; SUV: standardised uptake value; TEM: transmission electron microscopy imaging; TN: true negative; TP: true positive; T/t: tumour-to-tongue ratio; VOI: volume of interest.

2. DISCLAIMERS

Parts of this work has been previously presented as an abstract (ID #LB225) at the 2025 World Molecular Imaging Congress.

Valic M, Najjar E, Zheng M, et al. LB225- Porphyrin-lipid Nanotheranostics for Multimodal Imaging of Nodal Disease in Preclinical Oral Cancers. In: 2025 World Molecular Imaging Congress Program. Mol Imaging Biol. 2026; 28: 1–371.

3. SUPPLEMENTARY MATERIALS AND METHODS

3.1. Physicochemical nanoparticle characterisation

Measurement of ^{64}Cu-labelled PS samples was performed following storage at 2–8 °C until the sample radioactivity had completely decayed (after ~7 d). The morphology of ^{64}Cu-PS was visualised with transmission electron microscopy (TEM) (Tecnai G20; FEI, Hillsboro, USA) on formvar coated grids (FCF400-Cu-UB; Electron Microscopy Sciences, Hatfield, USA) using negative staining with 2.0 w/v% uranyl acetate (224002; Electron Microscopy Sciences). The hydrodynamic diameter and polydispersity index of ^{64}Cu-PS diluted in distilled water were measured in a glass cuvette (PCS1115; Malvern Panalytical, Westborough, USA) using dynamic light scattering with a 532-nm wavelength ‘green’ laser (Zetasizer Nano; Malvern Panalytical). The zeta (ζ)-potential of ^{64}Cu-PS diluted in distilled water was measured in a disposable folded capillary cell (DTS1070; Malvern Panalytical) using phase analysis light scatting.

3.2. Formula for ROC analysis

For calculating the diagnostic test performances of different imaging modalities for staging pN+ cervical lymph nodes from oral cancer models, the equations below were used:

(i) True positives (TP)

(ii) False negatives (FN)

(iii) False positive (FP)

(iv) True negative (TN)

(v) Sensitivity (SEN) = TP / Real positives

(vi) False negative rate (FNR) = FN / Real positives

(vii) False positive rate (FPR) = FP / Real negatives

(viii) Specificity (SPC) = TN / Real negatives

(ix) Accuracy (ACC) = (TP + TN) / (Real positives + Real negatives)

(x) Positive predictive value (PPV) = TP / (TP + FP)

(xi) Negative predictive value (NPV) = TN / (TN + FN)

(xii) Diagnostic odds ratio (DOR) = (SEN * SPC) / (FPR * FNR)

(xiii) F-score = (2 * PPV * TPR) / (PPV + TPR)

(xiv) Positive post-test probability = PPV

(xv) Negative post-test probability = 1 – NPV

4. SUPPLEMENTARY TABLES

Table S1. Summary of experimental details for animals used in study.

**Rat**	**Study I.D.**	**Weight (kg)**	**Tumour volume† (mL)**	**^{18}F-FDG dose (MBq/kg)**	**Blood glucose (mmol/L)**	**^{64}Cu-PS dose (MBq/kg)**	**RCP (%)**	**A_s (MBq/mg)**	**Pyro-lipid dose (mg/kg)**	**Route**	**pN+ staging prevalence**		
											Overall	L1 only	L2 only
#1	7894DE79	0.188	0.128	40	3.8	306	96	462	0.66	IV	67%	100%	0%
#2	7894DE7A	0.163	0.132	33	4.4	371	96	462	0.80	IV	17%	25%	0%
#3	7894DE7B	0.159	0.031	49	4.8	N/D	N/D	N/D	N/D	IV	50%	75%	0%
#4	7894DE7C	0.202	0.146	N/D	N/D	303	96	462	0.66	IV	17%	25%	0%
#5	78957479	0.229	0.121	42	5.4	171	92	314	0.55	IV	100%	100%	100%
#6	7895747A	0.195	0.058	36	4.8	223	92	314	0.71	IV	50%	75%	0%
#7	7895747B	0.225	0.170	47	5.8	198	92	314	0.63	IV	100%	100%	100%
#8	78A59FED	0.245	0.077	N/D	N/D	331	98	529	0.62	IT	100%	100%	100%
#9	78A59FEE	0.273	0.287	N/D	N/D	344	98	529	0.65	IT	67%	50%	100%
#10	78A59FEF	0.162	0.212	N/D	N/D	580	98	529	1.10	IT	50%	75%	0%
#11	78A59FF0	0.162	0.202	N/D	N/D	660	98	529	1.25	IT	33%	50%	0%
#12	78A59FF1	0.270	N/A	N/D	N/D	435	98	529	0.82	IT	N/A	N/A	N/A
#13	78A4964E	0.228	N/A	N/D	N/D	N/D	N/D	N/D	1.09	IV	N/A	N/A	N/A
#14	78A4964F	0.238	N/A	N/D	N/D	N/D	N/D	N/D	1.21	IV	N/A	N/A	N/A

Table S1. Summary of experimental details for animals used in study.

**Rat**	**Study I.D.**	**Weight (kg)**	**Tumour volume† (mL)**	**^{18}F-FDG dose (MBq/kg)**	**Blood glucose (mmol/L)**	**^{64}Cu-PS dose (MBq/kg)**	**RCP (%)**	**A_s (MBq/mg)**	**Pyro-lipid dose (mg/kg)**	**Route**	**pN+ staging prevalence**		
											Overall	L1 only	L2 only
#15	78B35EAD	0.208	0.319	49	4.9	613	97	585	1.05	IV	67%	75%	50%
#16	78B35EAE	0.198	0.231	51	5.6	545	97	585	0.93	IV	67%	75%	50%
#17	78B35EAF	0.214	0.172	61	3.6	493	97	585	0.84	IV	33%	25%	50%
#18	78B35EB1	0.202	0.216	53	5.6	570	97	585	0.97	IV	67%	75%	50%
#19	78B35EB2	0.246	N/A	N/D	N/D	320	97	585	0.55	IV	N/A	N/A	N/A
#20	78B35EB3	0.242	N/A	N/D	N/D	378	97	585	0.65	IV	N/A	N/A	N/A

†From MR imaging contours. N/A, not applicable; N/D, not done. N=4 rats used for model development are not included in Table.

Table S2. Activity of ^{64}Cu-PS at the injection site (tongue tumour) and in the tumour-draining cervical neck lymph nodes following IT injection. ^{64}Cu-PS: 80–120 MBq ^{64}Cu, 0.5 mg pyro-lipid, 0.05–0.10 mL, IT. Data derived from ^{64}Cu-PS PET image contours, decay-corrected to the time of injection. Means ± 1 S.D. Units %I.D. "Healthy" cohort are rats without tumours.

Timepoint post-IT injection (h)	Inj. Site (N=4)	Sentinel, all (N=8)	Sentinel, pN0 (N=3)	Sentinel, pN+ (N=5)	L1, all (N=15)	L1, pN0 (N=7)	L1, pN+ (N=8)	L2, all (N=8)	L2, pN0 (N=5)	L2, pN+ (N=3)	Healthy, all LNs (N=5)	Healthy, L1 LNs (N=2)	Healthy, L2 LNs (N=3)
1	58.9 ± 25.3	0.32 ± 0.23	0.32 ± 0.22	0.32 ± 0.26	0.36 ± 0.27	0.29 ± 0.27	0.42 ± 0.27	0.19 ± 0.10	0.13 ± 0.10	0.27 ± 0.08	0.09 ± 0.07	0.08 ± 0.01	0.10 ± 0.11
3	53.2 ± 17.6	0.42 ± 0.27	0.36 ± 0.28	0.45 ± 0.30	0.44 ± 0.33	0.37 ± 0.41	0.49 ± 0.27	0.26 ± 0.20	0.14 ± 0.09	0.32 ± 0.13	0.15 ± 0.12	0.18 ± 0.02	0.13 ± 0.17
6	50.5 ± 16.2	0.52 ± 0.34	0.44 ± 0.19	0.57 ± 0.41	0.54 ± 0.42	0.46 ± 0.49	0.60 ± 0.36	0.30 ± 0.22	0.19 ± 0.12	0.35 ± 0.18	0.21 ± 0.14	0.25 ± 0.09	0.18 ± 0.18
12	37.5 ± 8.1	0.56 ± 0.37	0.43 ± 0.12	0.64 ± 0.46	0.56 ± 0.39	0.45 ± 0.42	0.66 ± 0.37	0.41 ± 0.43	0.22 ± 0.12	0.35 ± 0.19	0.28 ± 0.17	0.31 ± 0.11	0.27 ± 0.22
24	28.2 ± 8.4	0.48 ± 0.31	0.35 ± 0.09	0.56 ± 0.38	0.48 ± 0.37	0.42 ± 0.45	0.54 ± 0.31	0.40 ± 0.55	0.17 ± 0.08	0.31 ± 0.17	0.29 ± 0.13	0.28 ± 0.06	0.30 ± 0.18
48	8.5 ± 2.2	0.38 ± 0.20	0.34 ± 0.07	0.41 ± 0.26	0.38 ± 0.28	0.36 ± 0.34	0.40 ± 0.24	0.35 ± 0.54	0.14 ± 0.08	0.24 ± 0.13	0.23 ± 0.09	0.24 ± 0.06	0.23 ± 0.12
72	7.1 ± 2.1	0.36 ± 0.22	0.26 ± 0.05	0.42 ± 0.27	0.39 ± 0.37	0.37 ± 0.50	0.41 ± 0.24	0.35 ± 0.56	0.12 ± 0.06	0.25 ± 0.13	0.13 ± 0.08	0.21 ± 0.06	0.08 ± 0.05

Table S3. Pharmacokinetics of IT injected ^{64}Cu-PS at the injection site (tongue tumour) and in the tumour-draining cervical neck lymph nodes. Means ± 1 S.D. Tukey's multiple comparisons t tests for pN0 *vs* pN+. α = 0.05. Post hoc power analysis of two independent means. "Healthy" cohort are rats without tumours.

		N	%I.D.$_{max}$ (%I.D.)	t$_{max}$ (h)	t$_{1/2}$ (h)	AUC$_{0-72h}$ (%I.D.*h)
**Injection site**		4	58.9 ± 25.3	1.0 ± 0.0	19.8 ± 45.0	7,917 ± 297
**Sentinel LNs**	All	8	0.59 ± 0.36	10.1 ± 6.6	89.7 ± 47.6	31.0 ± 18.4
	pN0	3	0.47 ± 0.19	7.0 ± 4.6	95.5 ± 117	24.8 ± 6.8
	pN+	5	0.66 ± 0.44	12.0 ± 7.3	86.2 ± 44.3	34.7 ± 22.9
	pN0 *vs* pN+ P value		> 0.99	> 0.99	> 0.99	> 0.99
	Power		0.10	0.14	0.05	0.09
**Level 1**	All	15	0.69 ± 0.54	11.1 ± 6.0	107 ± 35.2	37.2 ± 31.9
	pN0	7	0.49 ± 0.48	10.7 ± 6.9	177 ± 34.8	28.1 ± 29.4
	pN+	8	0.85 ± 0.56	11.3 ± 5.6	78.3 ± 50.7	44.3 ± 33.7
	pN0 *vs* pN+ P value		> 0.99	> 0.99	< 0.001 (***)	0.97
	Power		0.25	0.05	0.98	0.15
**Level 2**	All	8	0.28 ± 0.15	11.1 ± 4.3	233 ± 24.6	14.6 ± 8.6
	pN0	5	0.22 ± 0.12	12.0 ± 0.0	64.1 ± 53.7	11.2 ± 6.1
	pN+	3	0.37 ± 0.17	6.3 ± 5.5	103 ± 71.3	20.1 ± 10.5
	pN0 *vs* pN+ P value		> 0.99	> 0.99	0.34	> 0.99
	Power		0.05	0.54	0.12	0.26
**Healthy**	All	5	0.29 ± 0.13	19.2 ± 6.6	47.2 ± 48.5	16.4 ± 6.7
	L1	2	0.31 ± 0.11	18.0 ± 8.5	109 ± 129	17.8 ± 4.6
	L2	3	0.30 ± 0.18	20.0 ± 6.9	32.9 ± 34.6	15.5 ± 8.7

Multiplicity adjusted P values (Tukey's correction): not significant (ns) > 0.05, * ≤ 0.05, ** ≤ 0.01, *** ≤ 0.001.

Table S4. Uptake of ^{64}Cu-PS in the cervical neck lymph nodes of rats with tongue tumours and healthy rats 24 h post-IV injection. ^{64}Cu-PS: 250–500 MBq ^{64}Cu/kg, 0.5–1.0 mg/kg pyro-lipid, IV. Data derived from ^{64}Cu-PS PET image contours. Unitless. “Healthy” cohort are rats without tumours.

**Anatomical level & pathology**	**SUV_{mean}**				**SUV_{max}**			
	**Mean**	**Median**	**S.D.**	**N**	**Mean**	**Median**	**S.D.**	**N**
All pN0	1.17	1.09	0.68	25	1.89	1.69	0.91	25
All pN+	2.15	1.61	1.32	35	4.09	2.75	3.42	35
All healthy	0.92	0.87	0.30	12	1.62	1.54	0.36	12
L1a, pN0	1.80	1.82	0.53	3	3.03	3.56	1.01	3
L1a, pN+	2.77	2.62	1.26	17	5.36	4.98	2.74	17
L1a, healthy	1.25	1.26	0.18	4	1.62	1.49	0.35	4
L1b, pN0	1.51	1.29	0.66	10	2.04	1.80	0.78	10
L1b, pN+	1.92	1.27	1.20	10	3.79	2.49	2.65	10
L1b, healthy	0.75	0.71	0.24	4	1.76	1.86	0.44	4
L1, pN0	1.57	1.29	0.63	13	2.27	1.91	0.90	13
L1, pN+	2.46	2.56	1.32	27	4.78	4.67	2.76	27
L1, healthy	1.00	1.04	0.33	8	1.69	1.60	0.38	8
L2, pN0	0.73	0.63	0.40	12	1.48	1.23	0.76	12
L2, pN+	1.08	0.86	0.59	8	1.79	1.35	0.81	8
L2, healthy	0.75	0.77	0.11	4	1.47	1.46	0.30	4

Table S5. Statistical comparisons of ^{64}Cu-PS uptake in the cervical neck lymph nodes of rats with tongue tumours and healthy rats 24 h post-IV injection. Šídák multiple comparisons t tests. α = 0.05. Post hoc power analysis of two independent means. "Healthy" cohort are rats without tumours.

**Comparisons**	**SUV_{mean}**		**SUV_{max}**	
	**Adjusted P value**	**Power**	**Adjusted P value**	**Power**
All pN0 *vs* All pN+	0.02 (*)	0.916	0.004 (**)	0.868
All pN0 *vs* All healthy	>0.99	0.218	>0.99	0.160
All pN+ *vs* All healthy	0.03 (*)	0.875	0.02 (*)	0.680
L1a, pN0 *vs* L1a, pN+	>0.99	0.231	>0.99	0.272
L1a, pN0 *vs* L1a, healthy	>0.99	0.363	>0.99	0.572
L1a, pN+ *vs* L1a, healthy	0.48	0.611	0.08	0.718
L1b, pN0 *vs* L1b, pN+	>0.99	0.146	>0.99	0.475
L1b, pN0 *vs* L1b, healthy	>0.99	0.525	>0.99	0.094
L1b, pN+ *vs* L1b, healthy	>0.99	0.413	>0.99	0.278
L1, pN0 *vs* L1, pN+	0.57	0.610	0.02 (*)	0.873
L1, pN0 *vs* L1, healthy	>0.99	0.607	>0.99	0.371
L1, pN+ *vs* L1, healthy	0.03 (*)	0.846	0.02 (*)	0.858
L2, pN0 *vs* L2, pN+	>0.99	0.324	>0.99	0.131
L2, pN0 *vs* L2, healthy	>0.99	0.051	>0.99	0.050
L2, pN+ *vs* L2, healthy	>0.99	0.166	>0.99	0.104
L1a, pN0 *vs* L1b, pN0	>0.99	0.097	>0.99	0.172
L1a, pN0 *vs.* L1, pN0	>0.99	0.085	>0.99	0.227
L1a, pN0 *vs* L2, pN0	>0.99	0.953	>0.99	0.789
L1b, pN0 *vs* L1, pN0	>0.99	0.055	>0.99	0.094
L1b, pN0 *vs* L2, pN0	>0.99	0.902	>0.99	0.368
L1, pN0 *vs* L2, pN0	>0.99	0.965	>0.99	0.618
L1a, pN+ *vs* L1b, pN+	0.97	0.381	>0.99	0.288

Table S5. Statistical comparisons of ^{64}Cu-PS uptake in the cervical neck lymph nodes of rats with tongue tumours and healthy rats 24 h post-IV injection. Šídák multiple comparisons t tests. α = 0.05. Post hoc power analysis of two independent means. "Healthy" cohort are rats without tumours.

**Comparisons**	**SUV_{mean}**		**SUV_{max}**	
	**Adjusted P value**	**Power**	**Adjusted P value**	**Power**
L1a, pN+ *vs* L1, pN+	>0.99	0.117	>0.99	0.102
L1a, pN+ *vs* L2, pN+	0.010 (**)	0.929	0.004 (**)	0.928
L1b, pN+ *vs* L1, pN+	>0.99	0.196	>0.99	0.159
L1b, pN+ *vs* L2, pN+	>0.99	0.396	0.97	0.486
L1, pN+ *vs* L2, pN+	0.07	0.790	0.02 (*)	0.829
L1a, healthy *vs* L1b, healthy	>0.99	0.792	>0.99	0.988
L1a ,healthy *vs* L1, healthy	>0.99	0.243	>0.99	0.059
L1a, healthy *vs* L2, healthy	>0.99	0.974	>0.99	0.086
L1b, healthy *vs* L1, healthy	>0.99	0.227	>0.99	0.058
L1b, healthy *vs* L2, healthy	>0.99	0.050	>0.99	0.152
L1, healthy *vs* L2, healthy	>0.99	0.258	>0.99	0.149

Multiplicity adjusted P values (Šídák correction): not significant (ns) > 0.05, * ≤ 0.05, ** ≤ 0.01, *** ≤ 0.001.

1

Table S6. Uptake of ^{18}F-FDG in the cervical neck lymph nodes of rats with tongue tumours 45 min post-IV injection. ^{18}F-FDG: 46 MBq ^{18}F/kg, IV. Data derived from ^{18}F-FDG PET image contours. Unitless.

**Anatomical level & pathology**	**SUV_{mean}**				**SUV_{max}**			
	**Mean**	**Median**	**S.D.**	**N**	**Mean**	**Median**	**S.D.**	**N**
All pN0	2.50	2.91	1.44	23	4.41	3.62	2.72	23
All pN+	2.89	2.97	2.58	37	4.38	4.47	1.86	37
L1a, pN0	3.51	3.51	0.07	2	5.95	5.95	2.13	2
L1a, pN+	3.12	3.07	0.90	18	4.80	4.53	1.75	18
L1b, pN0	3.72	3.73	1.05	9	7.08	7.23	1.62	9
L1b, pN+	3.39	3.27	1.45	11	4.96	5.53	1.75	11
L1, pN0	3.68	3.57	0.94	11	6.88	7.23	1.66	11
L1, pN+	3.22	3.22	1.12	29	4.86	4.67	1.72	29
L2, pN0	1.42	1.02	0.83	12	2.14	1.99	0.80	12
L2, pN+	1.67	1.39	1.15	8	2.64	2.08	1.19	8

Table S7. Statistical comparisons of ^{18}F-FDG uptake in the cervical neck lymph nodes of rats with tongue tumours 45 min post-IV injection. Šídák multiple comparisons t tests. α = 0.05. Post hoc power analysis of two independent means.

**Comparisons**	**SUV_{mean}**		**SUV_{max}**	
	**Adjusted P value**	**Power**	**Adjusted P value**	**Power**
All pN0 *vs* All pN+	>0.99	0.100	>0.99	0.050
L1a, pN0 *vs* L1a, pN+	>0.99	0.088	>0.99	0.131
L1b, pN0 *vs* L1b, pN+	>0.99	0.084	0.44	0.750
L1, pN0 *vs* L1, pN+	>0.99	0.218	0.11	0.903
L2, pN0 *vs* L2, pN+	>0.99	0.099	>0.99	0.188
L1a, pN0 *vs* L1b, pN0	>0.99	0.057	>0.99	0.120
L1a, pN0 *vs* L1, pN0	>0.99	0.056	>0.99	0.099
L1a, pN0 *vs* L2, pN0	0.99	0.999	0.32	0.996
L1b, pN0 *vs* L1, pN0	>0.99	0.051	>0.99	0.058
L1b, pN0 *vs* L2, pN0	0.06	0.999	<0.001 (***)	1.00
L1, pN0 *vs* L2, pN0	0.04 (*)	0.999	<0.001 (***)	1.00
L1a, pN+ *vs* L1b, pN+	>0.99	0.092	>0.99	0.056
L1a, pN+ *vs* L1, pN+	>0.99	0.061	>0.99	0.052
L1a, pN+ *vs* L2, pN+	0.81	0.996	0.28	0.859
L1b, pN+ *vs* L1, pN+	>0.99	0.067	>0.99	0.053
L1b, pN+ *vs* L2, pN+	0.65	0.932	0.31	0.861
L1, pN+ *vs* L2, pN+	0.54	0.995	0.14	0.913

Multiplicity adjusted P values (Šídák correction): not significant (ns) > 0.05, * ≤ 0.05, ** ≤ 0.01, *** ≤ 0.001.

Table S8. In situ fluorescence of ^{64}Cu-PS in the cervical neck lymph nodes of rats with tongue tumours and healthy rats 24 h post-IV injection. ^{64}Cu-PS: 0.5–1.0 mg/kg pyro-lipid, IV. Relative fluorescence calculated from lymph node S/B ratio, where "background" is fluorescence intensity in mandibular glands. Unitless. "Healthy" cohort are rats without tumours.

Anatomical level & pathology	Mean relative fluorescence				Max relative fluorescence			
	**Mean**	**Median**	**S.D.**	**N**	**Mean**	**Median**	**S.D.**	**N**
All pN0	1.16	1.09	0.36	34	1.44	1.40	0.44	34
All pN+	1.91	1.81	0.68	44	2.13	2.14	0.64	44
All healthy	1.43	1.39	0.40	25	1.60	1.38	0.70	25
L1a, pN0	1.48	1.55	0.33	4	1.93	2.05	0.55	4
L1a, pN+	2.15	2.07	0.62	24	2.39	2.49	0.53	24
L1a, healthy	1.43	1.40	0.39	10	1.60	1.63	0.55	10
L1b, pN0	1.24	1.16	0.40	14	1.58	1.57	0.42	14
L1b, pN+	1.85	1.73	0.67	13	1.99	1.93	0.70	13
L1b, healthy	1.29	1.34	0.29	10	1.40	1.29	0.47	10
L1, pN0	1.29	1.18	0.39	18	1.66	1.75	0.46	18
L1, pN+	2.05	2.04	0.65	37	2.25	2.23	0.61	37
L1, healthy	1.36	1.38	0.34	20	1.50	1.41	0.51	20
L2, pN0	1.00	1.02	0.24	16	1.20	1.15	0.27	16
L2, pN+	1.18	1.18	0.25	7	1.50	1.52	0.31	7
L2, healthy	1.70	1.54	0.55	5	2.01	1.38	1.19	5

Table S9. Statistical comparisons of ^{64}Cu-PS in situ fluorescence in the cervical neck lymph nodes of rats with tongue tumours and healthy rats 24 h post-IV injection. Šídák multiple comparisons t tests. α = 0.05. Post-hoc power analysis of two independent means. "Healthy" cohort are rats without tumours.

**Comparisons**	**Mean relative fluorescence**		**Max relative fluorescence**	
	**Adjusted P value**	**Power**	**Adjusted P value**	**Power**
All pN0 *vs* All pN+	<0.001 (***)	0.999	<0.001 (***)	0.999
All pN0 *vs* All healthy	0.78	0.760	>0.99	0.185
All pN+ *vs* All healthy	0.02 (*)	0.888	0.02 (*)	0.883
L1a, pN0 *vs* L1a, pN+	0.48	0.521	>0.99	0.338
L1a, pN0 *vs* L1a, healthy	>0.99	0.055	>0.99	0.155
L1a, pN+ *vs* L1a, healthy	0.02 (*)	0.907	0.06	0.967
L1b, pN0 *vs* L1b, pN+	0.13	0.795	0.87	0.433
L1b, pN0 *vs* L1b, healthy	>0.99	0.062	>0.99	0.156
L1b, pN+ *vs* L1b, healthy	0.39	0.651	0.48	0.590
L1, pN0 *vs* L1, pN+	<0.001 (***)	0.994	0.03 (*)	0.945
L1, pN0 *vs* L1, healthy	>0.99	0.260	>0.99	0.166
L1, pN+ *vs* L1, healthy	<0.001	0.991	<0.001	0.996
L2, pN0 *vs* L2, pN+	>0.99	0.345	>0.99	0.610
L2, pN0 *vs* L2, healthy	0.34	0.975	0.26	0.711
L2, pN+ *vs* L2, healthy	0.92	0.522	0.97	0.231
L1a, pN0 *vs* L1b, pN0	>0.99	0.177	>0.99	0.255
L1a, pN0 *vs* L1, pN0	>0.99	0.138	>0.99	0.160
L1a, pN0 *vs* L2, pN0	0.94	0.884	0.59	0.959
L1b, pN0 *vs* L1, pN0	>0.99	0.064	>0.99	0.078
L1b, pN0 *vs* L2, pN0	>0.99	0.497	0.89	0.822
L1, pN0 *vs* L2, pN0	>0.99	0.703	0.56	0.924
L1a, pN+ *vs* L1b, pN+	0.91	0.265	0.93	0.477

Table S9. Statistical comparisons of ^{64}Cu-PS in situ fluorescence in the cervical neck lymph nodes of rats with tongue tumours and healthy rats 24 h post-IV injection. Šídák multiple comparisons t tests. α = 0.05. Post-hoc power analysis of two independent means. “Healthy” cohort are rats without tumours.

**Comparisons**	**Mean relative fluorescence**		**Max relative fluorescence**	
	**Adjusted P value**	**Power**	**Adjusted P value**	**Power**
L1a, pN+ *vs* L1, pN+	>0.99	0.090	>0.99	0.148
L1a, pN+ *vs* L2, pN+	0.001 (***)	0.972	0.06	0.982
L1b, pN+ *vs* L1, pN+	>0.99	0.153	0.98	0.239
L1b, pN+ *vs* L2, pN+	0.26	0.666	0.88	0.379
L1, pN+ *vs* L2, pN+	0.004 (**)	0.923	0.09	0.869
L1a, healthy *vs* L1b, healthy	>0.99	0.139	>0.99	0.131
L1a, healthy *vs* L1, healthy	>0.99	0.079	>0.99	0.076
L1a, healthy *vs* L2, healthy	>0.99	0.177	>0.99	0.396
L1b, healthy *vs* L1, healthy	>0.99	0.084	>0.99	0.079
L1b, healthy *vs* L2, healthy	0.98	0.430	0.82	0.270
L1, healthy *vs* L2, healthy	>0.99	0.395	0.90	0.302

Multiplicity adjusted P values: not significant (ns) > 0.05, * ≤ 0.05, ** ≤ 0.01, *** ≤ 0.001.

Table S10. Comparison of diagnostic test performances of different imaging modalities for staging pN+ cervical lymph nodes from oral cancer models using different cutoff point algorithms.

**Method**	Youden[a]							ROC01[b]						
**Modality**	Volume	^{64}Cu-PS PET		^{18}F-FDG PET		^{64}Cu-PS FL		Volume	^{64}Cu-PS PET		^{18}F-FDG PET		^{64}Cu-PS FL	
**Criterion**	mm^3	SUV_{mean}	SUV_{max}	SUV_{mean}	SUV_{max}	S/B_{mean}	S/B_{max}	mm^3	SUV_{mean}	SUV_{max}	SUV_{mean}	SUV_{max}	S/B_{mean}	S/B_{max}
**Cutoff**	≥ 10.1	≥ 2.15	≥ 1.74	≥ 2.11	≥ 2.54	≥ 1.35	≥ 1.87	≥ 13.81	≥ 1.09	≥ 1.74	≥ 2.11	≥ 3.62	≥ 1.35	≥ 1.87
**TP**	41	16	27	28	30	33	29	26	26	27	28	24	33	29
**FP**	28	2	8	12	13	7	6	19	12	8	12	11	7	6
**FN**	11	19	8	9	7	11	15	26	9	8	9	13	11	15
**TN**	10	23	17	11	10	27	28	19	13	17	11	12	27	28
**SEN**	79%	46%	77%	76%	81%	75%	66%	50%	74%	77%	76%	65%	75%	66%
**FPR**	74%	8%	32%	52%	57%	21%	18%	50%	48%	32%	52%	48%	21%	18%
**FNR**	21%	54%	23%	24%	19%	25%	34%	50%	26%	23%	24%	35%	25%	34%
**SPC**	26%	92%	68%	48%	43%	79%	82%	50%	52%	66%	48%	52%	79%	82%
**PPV**	59%	89%	77%	70%	70%	83%	83%	58%	68%	77%	70%	69%	83%	83%
**NPV**	48%	55%	68%	55%	59%	71%	65%	42%	59%	68%	55%	48%	71%	65%
**ACC**	57%	65%	73%	65%	67%	77%	73%	50%	65%	73%	65%	60%	77%	73%
**F-score**	68%	60%	77%	73%	75%	79%	73%	54%	71%	77%	73%	67%	79%	73%
**DOR**	1.3	9.7	7.2	2.9	3.3	11.6	9.0	1.0	3.1	7.2	2.9	2.0	11.6	9.0

[a]Cutoff point algorithm maximising difference between the true positive rate (or sensitivity) and false positive rate (1-specificity). [b]Cutoff point algorithm minimising the distance between ROC curve and point (0,1).

1

Table S10. Comparison of diagnostic test performances of different imaging modalities for staging pN+ cervical lymph nodes from oral cancer models using different cutoff point algorithms.

**Method**	MaxDOR[c]							MinValueNPV[d]						
**Modality**	Volume	^{64}Cu-PS PET		^{18}F-FDG PET		^{64}Cu-PS FL		Volume	^{64}Cu-PS PET		^{18}F-FDG PET		^{64}Cu-PS FL	
**Criterion**	mm^3	SUV_{mean}	SUV_{max}	SUV_{mean}	SUV_{max}	S/B_{mean}	S/B_{max}	mm^3	SUV_{mean}	SUV_{max}	SUV_{mean}	SUV_{max}	S/B_{mean}	S/B_{max}
**Cutoff**	≥ 4.94	≥ 2.46	≥ 1.29	≥ 2.11	≥ 2.54	≥ 1.81	≥ 2.24	≥ 4.94	≥ 0.39	≥ 1.29	≥ 1.10	≥ 2.54	≥ 1.02	≥ 1.17
**TP**	51	14	33	28	30	21	18	51	35	33	31	30	42	42
**FP**	36	1	15	12	13	1	1	36	19	15	15	13	20	22
**FN**	1	21	2	9	7	23	26	1	0	2	6	7	2	2
**TN**	2	24	10	11	10	33	33	2	6	10	8	10	14	12
**SEN**	98%	40%	94%	76%	81%	48%	41%	98%	100%	94%	84%	81%	95%	95%
**FPR**	95%	4%	60%	52%	57%	3%	3%	95%	76%	60%	65%	57%	59%	65%
**FNR**	2%	60%	6%	24%	19%	52%	59%	2%	0%	6%	16%	19%	5%	5%
**SPC**	5%	96%	40%	48%	43%	97%	97%	5%	24%	40%	35%	43%	41%	35%
**PPV**	59%	93%	69%	70%	70%	95%	95%	59%	65%	69%	67%	70%	68%	66%
**NPV**	67%	53%	83%	55%	59%	59%	56%	**67%**	**100%**	**83%**	**57%**	**59%**	**88%**	**86%**
**ACC**	59%	63%	72%	65%	67%	69%	65%	59%	68%	72%	65%	67%	72%	69%
**F-score**	73%	56%	80%	73%	75%	64%	57%	73%	79%	80%	75%	75%	79%	78%
**DOR**	**2.8**	**16.0**	**11.0**	**2.9**	**3.3**	**30.1**	**22.8**	2.8	N/A	11.0	2.8	3.3	14.7	11.5

[c]Cutoff point algorithm maximising the DOR. [d]Cutoff point algorithm fulfilling the condition NPV ≥80%.

5. SUPPLEMENTARY FIGURES

Supplementary Figure S1

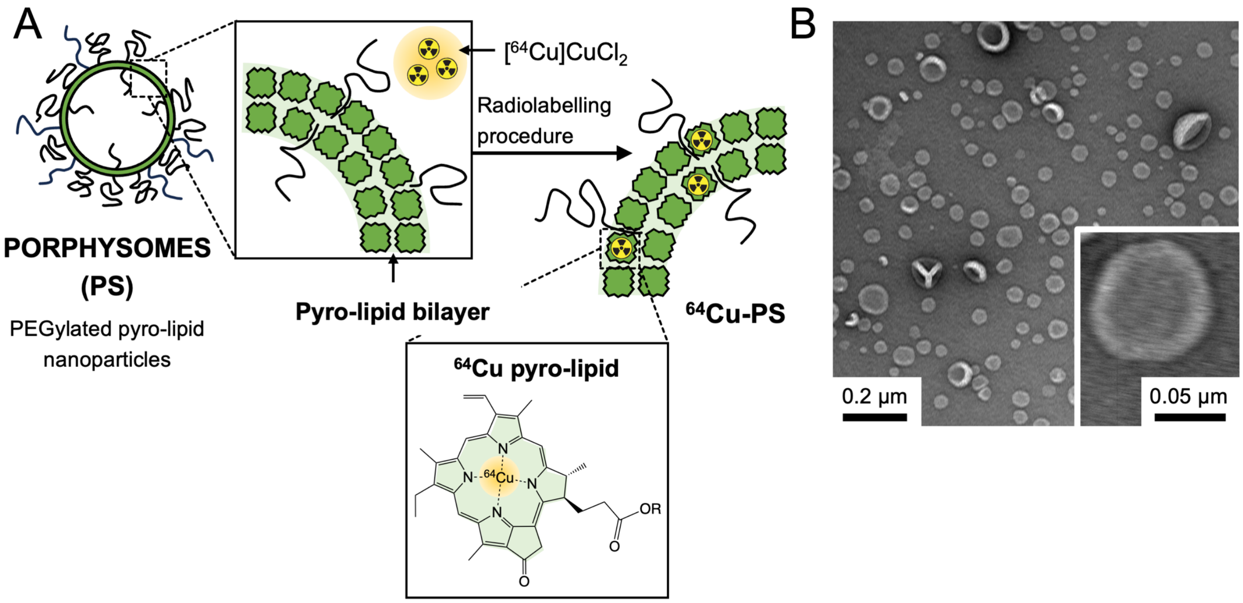

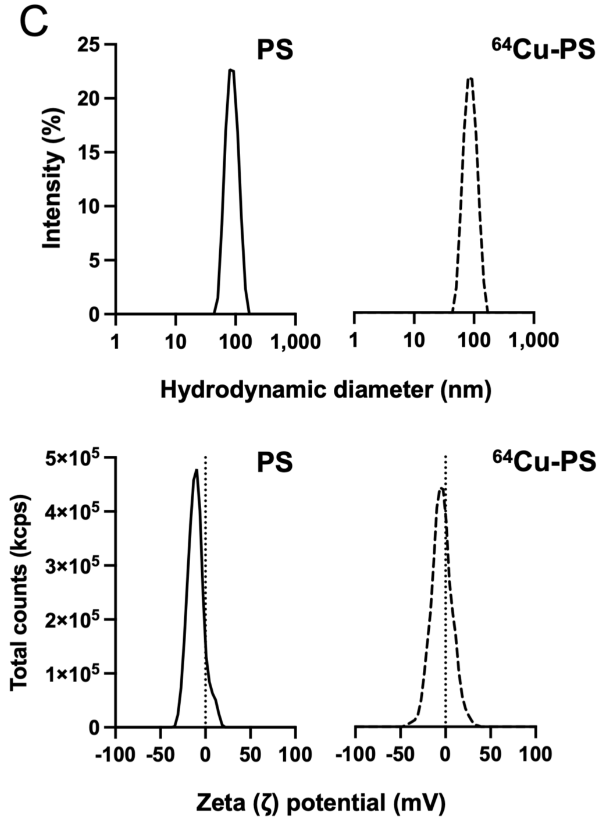

DLS parameters	PS	^{64}Cu-PS
Size	84.9 ± 0.5 nm	85.7 ± 2.6 nm
PDI	0.05 ± 0.01	0.09 ± 0.02
ζ potential	-10.3 ± 3.84 mV	-3.54 ± 0.60 mV

Mean ± 1 S.D.

Figure S1. Preparation of ^{64}Cu-labelled PS nanotheranostics. (A) 'One pot' labelling scheme for preparing ^{64}Cu-PS. Approximately ~10^{-2} mol% of all pyro-lipid building blocks are chelated to ^{64}Cu radiometals (or ~5 out of 80,000 pyro-lipids per PS nanoparticle). (B) Morphology of ^{64}Cu-PS from TEM imaging. (C) ^{64}Cu-PS particle size (hydrodynamic diameter) and PDI from DLS measurements. Zeta potential from PALS measurements.

Supplementary Figure S2

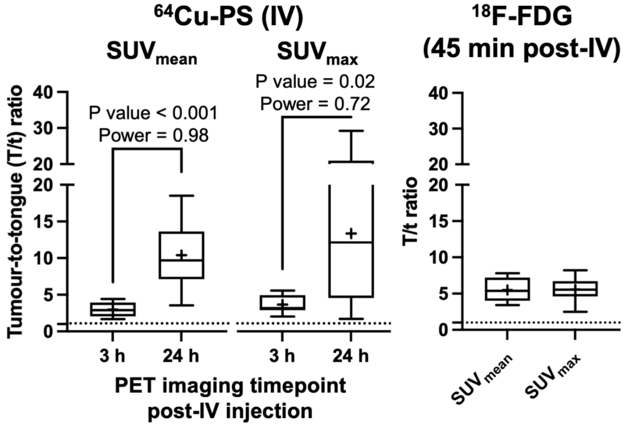

	PET ROIs	SUV$_{mean}$	SUV$_{max}$
Tumour uptake	^{18}F-FDG	5.24 ± 1.82	10.2 ± 3.24
	^{64}Cu-PS 3 h	2.41 ± 0.73	4.66 ± 1.58
	^{64}Cu-PS 24 h	4.39 ± 1.80	12.4 ± 7.60
T/t ratio	^{18}F-FDG	5.5 ± 1.6	5.6 ± 1.6
	^{64}Cu-PS 3 h	3.0 ± 1.0	3.7 ± 1.2
	^{64}Cu-PS 24 h	10.4 ± 4.5	13.4 ± 9.3

Mean ± 1 S.D.

Figure S2. SUVs and tumour-to-tongue (T/t) ratios from ^{64}Cu-PS and ^{18}F-FDG PET images. ^{18}F-FDG PET/MR images from 45 min post-IV injection (46 MBq ^{18}F/kg), and ^{64}Cu-PS PET/MR images from 3 h and 24 h post-IV injection (250–500 MBq ^{64}Cu/kg, 0.5–1.0 mg/kg pyro-lipid). Means ± 1 S.D. SUVs and T/t ratios unitless. Statistics compare SUVs at 3 h *vs* 24 h using multiple comparisons t tests (Tukey correction) and $\alpha = 0.05$. Power analysis is a post hoc test of two independent means.

Supplementary Figure S3

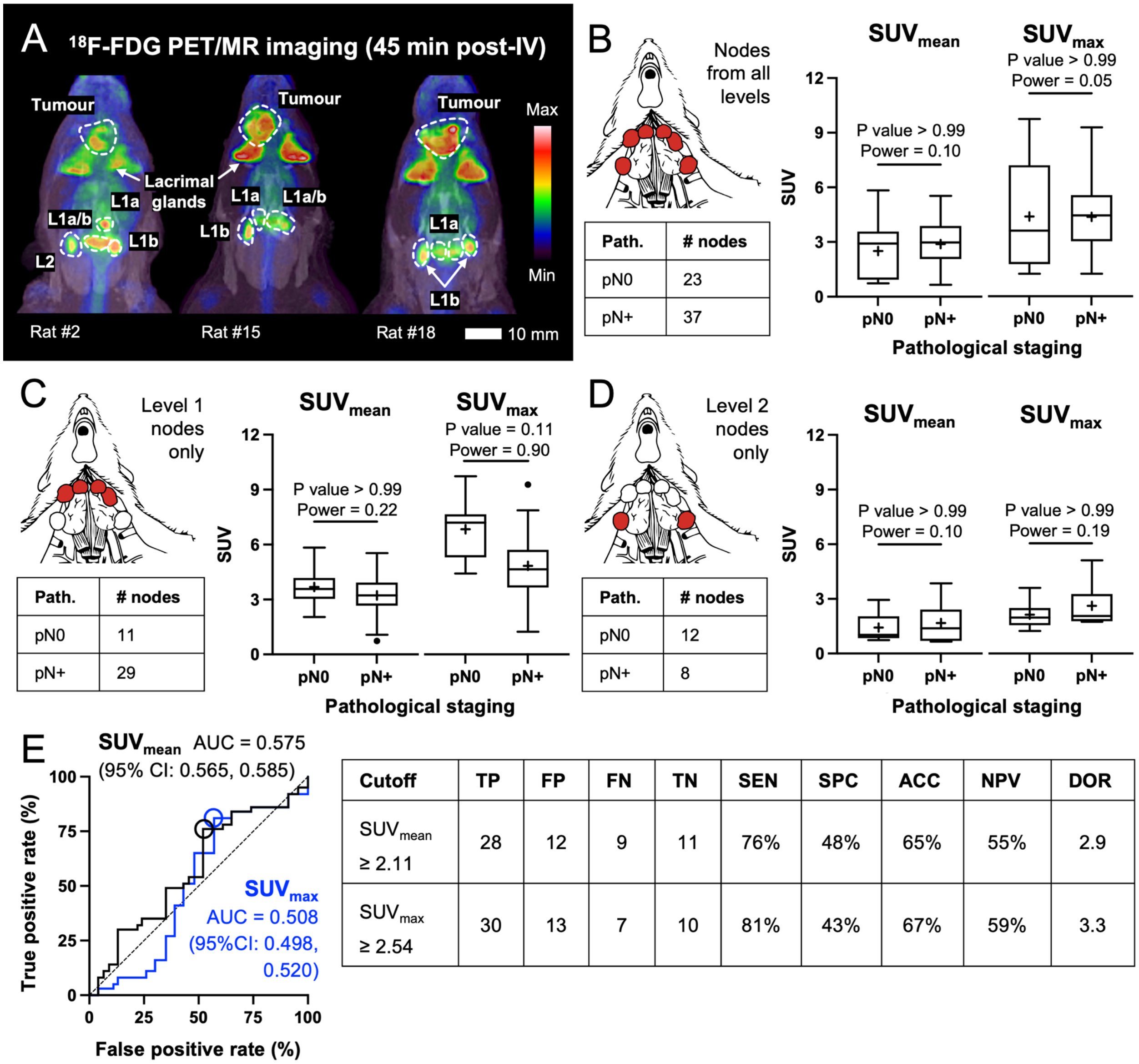

Cutoff	TP	FP	FN	TN	SEN	SPC	ACC	NPV	DOR
SUV_{mean} ≥ 2.11	28	12	9	11	76%	48%	65%	55%	2.9
SUV_{max} ≥ 2.54	30	13	7	10	81%	43%	67%	59%	3.3

Figure S3. PET imaging of ^{18}F-FDG radiotracer uptake in neck lymph nodes of tongue tumour model. (A) Representative MIPs of ^{18}F-FDG PET/MR images from 45 min post-IV injection (46 MBq ^{18}F/kg). ^{18}F-FDG uptake in pN0 *vs* pN+ staged nodes from (B) all anatomical levels, (C) level 1 only, and (D) level 2 only. Tukey box-and-whisker plots with "+" at mean. SUVs unitless. Statistics compare pN0 *vs* pN+ staged nodes using multiple comparisons t tests (Tukey correction) and α = 0.05. Power analysis is a post hoc test of two independent means. (E) ROC curves and diagnostic performance of tests predicting pN+ staged nodes using PET imaging SUV. ROC AUCs and 95% CIs listed in brackets.

Supplementary Figure S4

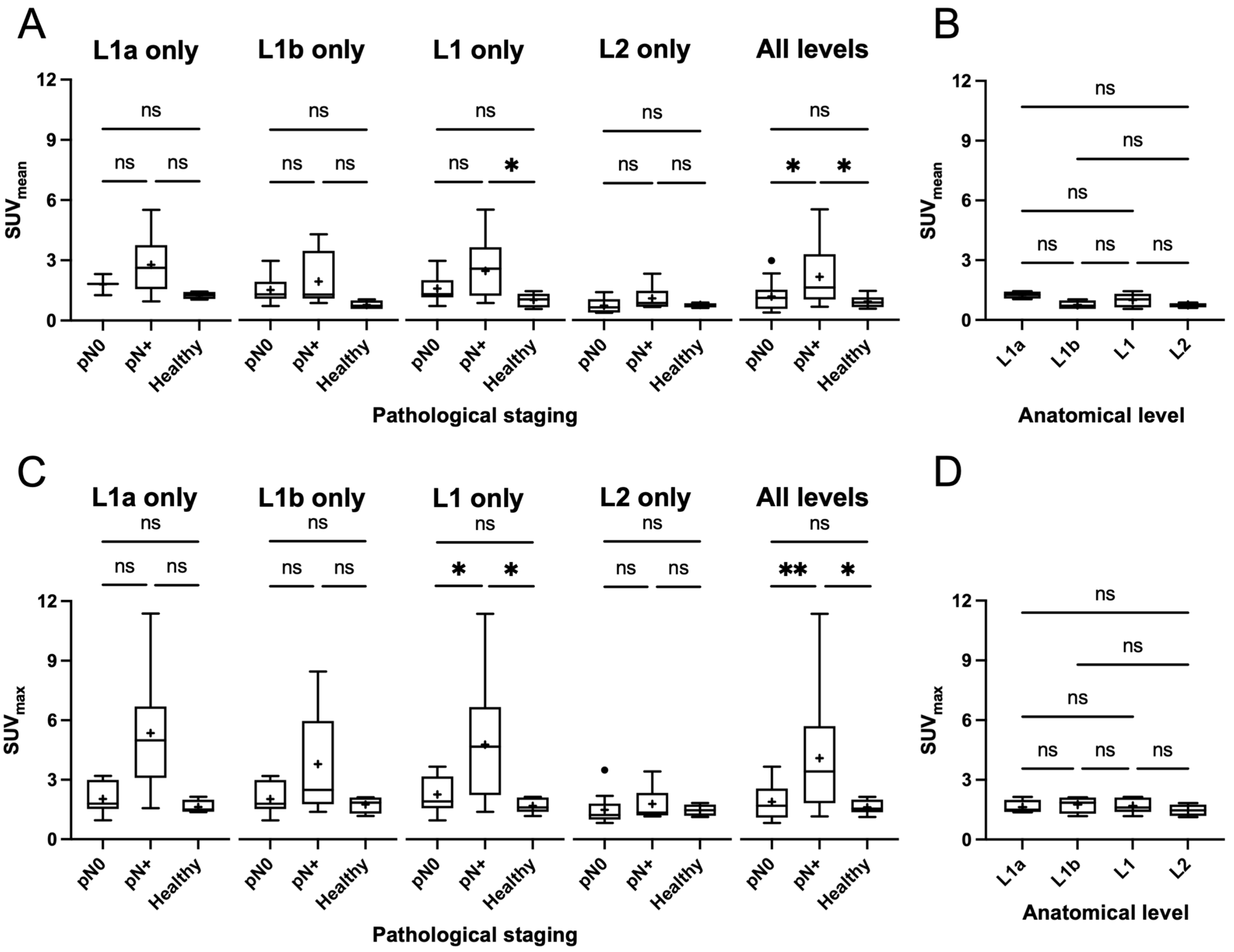

E

Level	# nodes		
	pN0	pN+	Healthy
L1a	3	17	4
L1b	10	10	4
L1	13	27	8
L2	12	8	4
All	25	35	12

Figure S4. Level-by-level analysis of ^{64}Cu-PS uptake in neck lymph nodes from PET/MR imaging. (A) SUV_{mean} and (C) SUV_{max} of ^{64}Cu-PS (250–500 MBq ^{64}Cu/kg, 0.5–1.0 mg/kg pyro-lipid) 24 h post-IV injection in pN0 and pN+ staged nodes of oral cancer models, and in healthy nodes from rats without tongue tumours. (B, D) Uptake analysis in cervical lymph nodes from "healthy" rats without tumours. Tukey box-

and-whisker plots with “+” at mean. SUVs unitless. Statistics: multiple comparisons t tests (Tukey correction) and $\alpha = 0.05$. Multiplicity adjusted P values: not significant (ns) > 0.05, * ≤ 0.05, ** ≤ 0.01, *** ≤ 0.001. (E) Anatomical distribution and pathological staging of neck nodes from oral cancer models and healthy rats without tumours.

Supplementary Figure S5

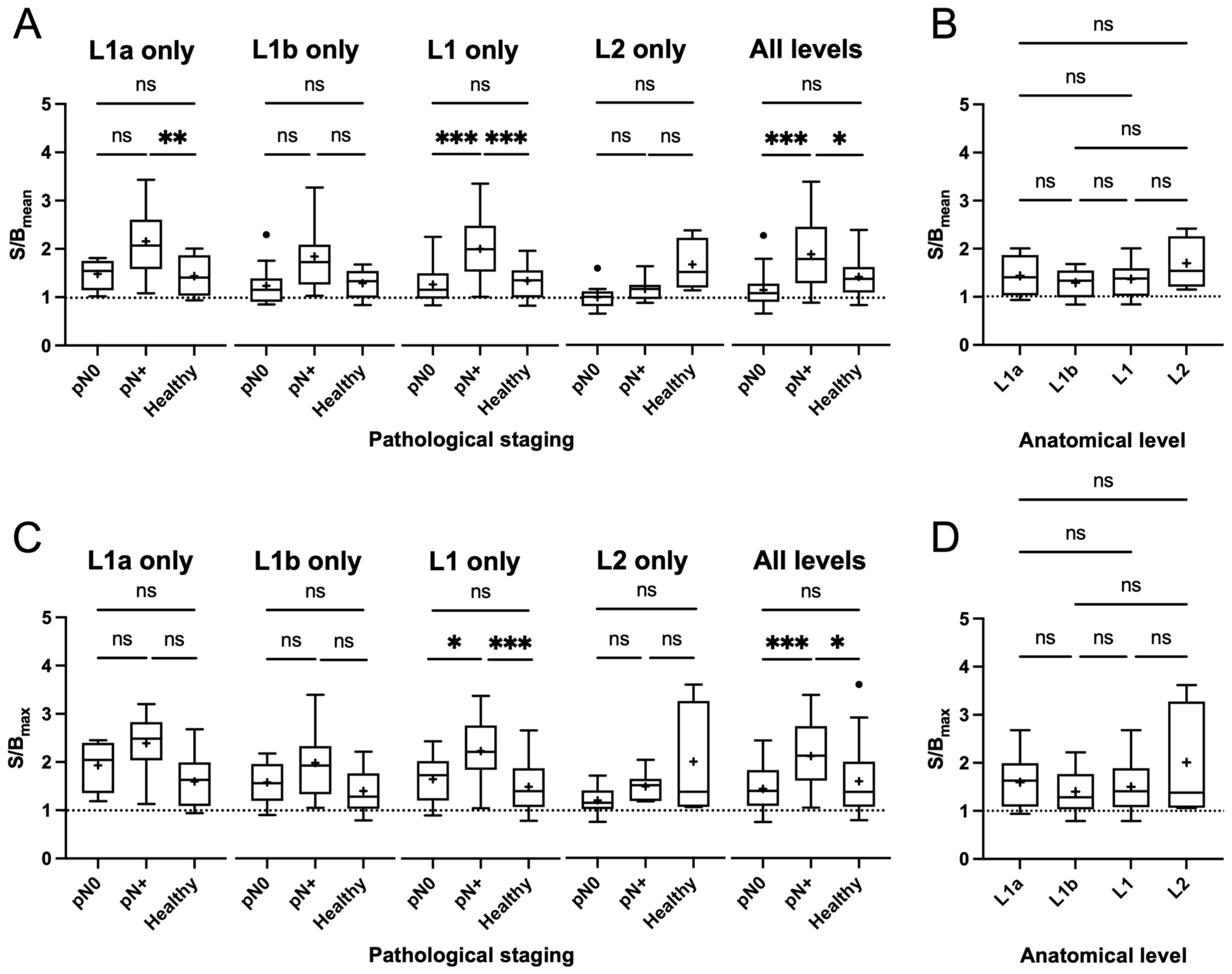

E

Level	# nodes		
	pN0	pN+	Healthy
L1a	4	24	10
L1b	14	13	10
L1	18	37	20
L2	16	7	5
All	44	34	25

Figure S5. Level-by-level analysis of ^{64}Cu-PS relative fluorescence in neck lymph nodes from in situ FL imaging. (A) Signal-to-background $(S/B)_{mean}$ and (C) S/B_{max} ratios of PS (0.5–1.0 mg/kg pyro-lipid) 24 h post-IV injection in pN0 and pN+ staged lymph nodes of oral cancer models, and in healthy nodes from rats without tongue tumours. (B, D) Relative fluorescence analysis in cervical lymph nodes from only

"healthy" rats without tumours. Tukey box-and-whisker plots with "+" at mean. S/B ratios unitless. Statistics: multiple comparisons t tests (Tukey correction) and $\alpha = 0.05$. Multiplicity adjusted P values: not significant (ns) > 0.05, * ≤ 0.05, ** ≤ 0.01, *** ≤ 0.001. (E) Anatomical distribution and pathological staging of neck nodes from oral cancer models and healthy rats without tumours.
